# Supplementary material for: Near-Perfect Synaptic Integration by Nav1.7 in Hypothalamic Neurons Regulates Body Weight
Source: Cell. 2016 Jun 16;165(7):1749–61. doi: 10.1016/j.cell.2016.05.019 (PMC4912688; doi:10.1016/j.cell.2016.05.019)
Supplement: Document S1. Supplemental Experimental Procedures [file mmc1.pdf]

Cell, Volume *165*

Supplemental Information

Near-Perfect Synaptic Integration by Na_v1.7 in Hypothalamic Neurons Regulates Body Weight

Tiago Branco, Adam Tozer, Christopher J. Magnus, Ken Sugino, Shinsuke Tanaka, Albert K. Lee, John N. Wood, and Scott M. Sternson

Supplemental Experimental Procedures

All experimental protocols were conducted according to U.S. National Institutes of Health guidelines for animal research and approved by the Institutional Animal Care and Use Committee at Janelia Research Campus. Experiments conducted in the UK were licensed under the UK Animals (Scientific Procedures) Act of 1986 following local ethical approval.

Mice. Mice were housed on a 06:00-18:00h light cycle with water and mouse chow *ad libitum* (PicoLab Rodent Diet 20, 5053 tablet, TestDiet) unless otherwise noted. Adult male mice (>6 weeks old) were used for experiments. Cre recombinase-expressing lines were used: *Npy*^{hrGFP} (Jackson Labs Stock 006417), *Pomc*^{topazFP} (Jackson Labs Stock 008322), *Agrp*^{Cre} (Jackson Labs Stock 012899, *Agrp*$^{tm1(cre)Lowl}$/J), *Pomc*^{Cre} (BAC transgenic provided by Dr. Joel Elmquist, University of Texas Southwestern Medical Center), *Scn9a*$^{tm1.1Jnw}$ (*Scn9a*$^{flox/flox}$, J.N.W., University College London) (Weiss et al., 2011). To generate *Agrp*$^{Cre/+;}$*Scn9a*$^{flox/flox}$ or *Pomc*$^{Cre/+}$*;Scn9a*$^{flox/flox}$, homozygous *Scn9a*$^{flox/flox}$ mice were crossed to either *Agrp*^{Cre} or *Pomc*^{Cre} and the double heterozygous progeny were then crossed with homozygous *Scn9a*$^{flox/flox}$. *Agrp*$^{Cre/+;}$*Scn9*$^{flox/flox-}$ or *Pomc*$^{Cre/+;}$*Scn9a*$^{flox/flox}$ mice were identified by genotyping tail DNA; in addition the possibility for global *Scn9a* deletion was monitored by including a primer for the presence of the knock-out allele in the genotyping PCR reaction, which was negative for all mice included in the study. For tail DNA from a subset of animals we also sequenced the genotyping band and confirmed that the loxP flanked exons 14 and 15 of *Scn9a* were intact.

Recombinant adeno-associated viral (rAAV) vectors. The following viral vectors (Atasoy et al., 2008) were used in this study: rAAV2/9-*CAG::FLEX-rev-hrGFP:miR30(Scn9a)* and rAAV2/9-*CAG::FLEX-rev-hrGFP:miR30(scrambled-Scn9a)* (1.5 e13 - 1.7e13 Genomic Copies (GC)/ml, Janelia, http://www.addgene.org/Scott_Sternson/), rAAV2/9-*CAG*-FLEX-EGFP (7e12 GC/ml, Penn), *rAAV9.hSyn.HI.eGFP-Cre* (7e12GC/ml,Penn), *rAAV9.eF1a.dflox.ChR2(H134R).mCherry*

(7e12 GC/ml, Penn) *CAG*: promoter containing a cytomegalovirus enhancer; chicken beta actin promoter, first exon, and first intron; and the splice acceptor of rabbit beta-globin gene. FLEX: Cre-dependent flip-excision switch (Atasoy et al., 2008).

Viral injections were performed as described previously (Betley et al., 2013). Bilateral ARC viral injections in *Agrp*^{Cre} *or Pomc*^{Cre} mice were made at two depths using the following coordinates: bregma: -1.7 mm; midline: ±0.24 mm; dorsal surface: -5.95 mm (100 nl/site). Coordinates for bilateral PVH injection in *Scn9a*$^{flox/flox}$ mice were: bregma -0.85mm; midline -0.22mm; dorsal surface -4.77mm (40 nl/site). Mice were used for electrophysiology 8-12 days post infection.

RNA sequencing. AGRP neurons were manually sorted from the ARC of *Npy*^{hrGFP} mice using methods described previously (Hempel et al., 2007). The full RNA-Seq data set has been recently reported (Henry et al., 2015).

Fluorescent in situ hybridization. Two-color FISH was performed on hypothalamus-containing fixed frozen sections from male *Agrp*^{Cre} mice (8-9 weeks old), using the proprietary probes and methods of Advanced Cell Diagnostics (ACD Technical notes #320535 for tissue prep, and #320293 for Multiplex labeling, http://www.acdbio.com/technical-support/downloads). Briefly animals were anesthetized and sequentially perfused with RNase free solutions of PBS and 4% paraformaldehyde (PFA) in phosphate buffered saline (PBS). The brains were removed and post-fixed (24 h, 4 ºC) in 4% PFA in PBS, incubated in 30% sucrose (12 h), and the blocked brain was mounted in cryo-embedding media (OCT) on a cryostat for sectioning. Frozen sections (15 µm) were mounted on slides, which were air dried (20 min at -20 ºC) or stored -80 ºC for later use. The OCT was washed off with PBS before pretreatment with ACD proprietary reagents PT2 & PT4. After boiling for 5 min in PT2, sections were rinsed in distilled water then ethanol, air dried, and then incubated with ACD proprietary reagent PT4 (30 min, 40 ºC) in a

HybEZ sealed humidified incubator (Advanced Cell Diagnostics). We performed dual probe labeling, using probes for *Scn9a* in Channel 1 (Mm-Scn9-C1, #313341-C1), and either *Agrp* (Mm-Agrp-C2, #400711-C2) or *Pomc* (Mm-Pomc-C2, #314081-C2) in Channel 2. Probes were mixed at a 1:50 ratio of Channel 2 and Channel 1 probes. Wax-outlined tissue sections were immersed in Probe mix and incubated (2 h, 40 ºC) in the HybEZ humidified incubator, rinsed in ACD Wash Buffer (2 x 2') then sequentially incubated in ACD proprietary reagents alternating AMP1-FL and AMP3-FL (30 min) with AMP2-FL and AMP4-FL (15 min) with 2 washes (2 min) between each step. Brain sections were then labeled with DAPI and coverslips were applied. Slides were stored at 4 ºC before image acquisition at 10×, 20×, and 63× using a Zeiss 710 confocal on an Axio Examiner Z1 upright microscope. Quantification of *Scn9a* particles (Fig. 2F) was performed on single confocal optical sections acquired with a 63× objective, using StarSeach (http://rajlab.seas.upenn.edu/StarSearch/launch.html) with a threshold setting of 50. For colocalization of *Scn9a* with *Agrp* or *Pomc*, we used single confocal optical sections (acquired with a 63× objective); for the small number of cells that did not show colocalization in a single optical section AGRP: (3/328 neurons, POMC: 2/169 neurons) we examined other optical sections for the neuron and classified as co-expressing if we identified *Scn9a* elsewhere in the neuron (colocalization detected in 3/5 of these neurons).

Constructs for *Scn9a* knockdown. *miR30*-based shRNA constructs for *Scn9a* were developed using miR_Scan software (http://www.ncbi.nlm.nih.gov/staff/ogurtsov/projects/mi30/) (Matveeva et al., 2012) with the *Scn9a* coding sequence (NM_01290674.1, position: 3728-3748). Using miR_Scan and associated rules for *miR30*-based shRNA (Dow et al., 2012; Matveeva et al., 2012), we selected the sense strand sequence CGGGUGGCAUACGGGUAUAAAA (based on design rules (Dow et al., 2012), the 5' base of shRNA sense strand is not homologous to the coding sequence) and the guide strand sequence UUUUAUACCCGUAUGCCACCCA (miR_Scan score: 84.6, sequence position: 3727-3748). As

an additional criterion, we looked for homology to mouse RefSeq RNA <80%, and for established RefSeq mRNA in the mouse, the closest homology sequences, other than *Scn9a*, were Olfr372 (61% identity) and Traf3 (57% identity). A *miR30* construct based on previously described sequence (Stern et al., 2008) was synthesized with sense and guide strand sequences from *Scn9a* and was placed in the 3'UTR of a hrGFP expression vector in pcDNA3.1. To produce a negative control for this *miR30*-based *Scn9a* shRNA construct, we used an online tool to produce a scrambled sequences (http://www.sirnawizard.com/scrambled.php) and then chose a sequence with <76% homology to RefSeq transcripts in the mouse genome and that also obeyed guidelines for *miR30*-based shRNA (Dow et al., 2012; Matveeva et al., 2012) (sense strand: ACGAAGTCAGGGAGGAATTTAT; guide strand: AUAAAUUCCUCCCUGACUUCGC). The sequences were subcloned into pcDNA3.1. After establishing effective knockdown of *Scn9a* in HEK cells (see below) with *hrGFP-miR30(Scn9a)* but not *hrGFP-miR30(scambled-Scn9a)*, we placed these sequences into an rAAV vector between a FLEX switch sequence (Atasoy et al., 2008) downstream of a *CAG* promoter in an rAAV2 vector.

HEK cell electrophysiology. Constructs for shRNA were characterized in whole cell voltage clamp experiments performed on HEK293 cells stably expressing murine *Scn9a*. Cells were plated at low density ($4\times10^3/cm^2$) on poly-D-lysine coated glass coverslips in 10 cm^2 wells, then transfected using the Fugene 6 transfection reagent (Roche) with a DNA plasmid (pcDNA3.1) with *hrGFP-miR30(Scn9a)* or *hrGFP-miR30(scrambled-Scn9a)* (2 μg per well), followed by replacement of media after 3 h. Recordings were made 24-72 h post-transfection. Patch clamp recordings were made 24-72 h post-transfection in whole cell voltage clamp on an inverted amplifier (Molecular Devices CA). Electrophysiological data was filtered at 5 kHz, digitized at 20 kHz using a Digidata 1400 interface (Molecular Devices CA), recorded on a PC (Superlogics), and analysed using custom Matlab software (MathWorks). Electrode resistance was 2-3 MOhm and 85-95% series resistance compensation was routinely applied. Cells were continuously

perfused from a gravity fed perfusion system. External solution contained (mM): NaCl 140, HEPES 10, Glucose 5, KCl 4, $CaCl_2$ 2, $MgCl_2$ 1. Whole cell pipette internal solution contained (mM); CsCl 31, CsGluconate 87, NaCl 5, HEPES 10, EGTA 10 MgATP 3. Osmolarity was within 280-300 mOsm/L, and pH was adjusted using CsOH to within 7.3 - 7.4.

Electrophysiology in brain slices. Acute coronal slices (200 μm) were prepared at the level of the ARC from male mice (5-10 weeks) expressing a viral vector or fluorescent proteins in AGRP or POMC neurons. Animals were killed by decapitation following isoflurane anaesthesia. Coronal slices were cut at 4°C using a 7000smz-2 vibrating microtome (Campden, UK). Brain slices were incubated at 37°C for one hour before being kept at room temperature prior to experiments, in artificial cerebrospinal fluid (ACSF) containing (in mM): $NaCl_2$ 125, KCl 2.5, $NaHCO_3$ 26, NaH_2PO_4 1.25, glucose 25, $CaCl_2$ 2, $MgCl_2$ 1 (pH 7.3 when bubbled with 95% O_2 and 5% CO_2). Boroscillicate glass micropipettes with a 3-6 MΩ resistance (Harvard Apparatus, UK) were filled with (in mM): K-Gluconate 130, KCl 10, HEPES 10, EGTA 1, Na_2ATP 2, Mg_2ATP 2, Na_2GTP 0.3, filtered (2 μm) prior to patching. For cell-attached recordings pipettes were filled with ACSF and currents recorded in voltage-clamp with a holding current of 0 pA. EPSCs were recorded at a holding potential of -70 mV. Transient sodium currents were elicited with a 50 ms voltage step to 0 mV from -80 mV. Fluorescent cells were visualized on an upright Slicescope (Scientifica, UK) using a 60× objective. Somatic whole-cell patch clamp recordings (R_{series} <30 MΩ) were achieved using a HEKA 800 Amplifier (HEKA, Germany). Data was acquired at 25-50 kHz using custom software and filtered at 8 kHz. All voltage recordings were obtained with a seal resistance > 5 GΩ and within 10 minutes of gaining whole-cell access, and cells were discarded if there was more than a 10% change in input resistance. All recordings were performed at 35-37°C.

***In vivo* electrophysiology.** *In vivo* whole cell patch clamp recordings, were made from control and virally transduced *miR30(Scn9a)* knockdown in *Sim1*^{Cre} mice in the PVH. Mice were

sequentially anesthetized with 2% isoflurane, which was replaced by a longer-term intraperitoneal anesthetic mix, comprised of Fentanyl (0.0075 mpk), Dexdomitor (0.112 mpk), and Midazolam (6 mpk), which was supplemented with doses of 1/3 the initial volume every 45 minutes (Lee et al., 2012). Mice were secured in a stereotaxic frame, local anesthetic was applied (marcaine, 2 mg/kg), a small (diameter <1.0 mm) craniotomy was drilled in the skull over the PVH (~-0.8 mm A/P and +0.2 mm M/L) and the dura was removed.

Due to the physical resistances involved in traversing almost 5 mm of brain with a patch pipette in this region, we found that best results were obtained by perforating a pathway to 4.6 mm below the surface of the cortex, nearly to the level of the PVH, which was followed by introducing the recording pipet via this lower resistance pathway. The perforation was performed with a patch electrode similar to those used for recordings, but broken back to 50 µm diameter at the tip and sharpened by beveling at ~35°. This pipette was filled with the same HEPES buffered ACSF used in our HEK cell recordings. No positive air pressure was applied to the pipette during perforation.

In vivo whole-cell recordings were made after using positive air pressure to maintain a clear pipette tip during descent (surface→-4.5 mm: 900 mB, -4.5 mm→-4.6 mm: 200 mB, -4.6 mm→-4.7 mm: 50 mB, then 15-20 mB while stepping at 1 µm steps through the PVH (PVH was at -4.7 mm to -4.9 mm from cortical surface, calibrated with fluorescent bead injections in pilot experiments) until contact with a cell was detected by a change in the pipette tip resistance, and a gigaseal formed. Patch electrodes (4-10 MOhm) contained 125 mM Potassium Gluconate, 11 mM Potassium Chloride, 10 mM HEPES, 10 mM Phosphocreatine, 4 mm ATP, 0.3 mM GTP, and 2.5 mM Biocytin, pH 7.3 and 285 mOsm. Data was recorded on a HEKA Patchmaster system, sampled at 50 kHz after filtering at 10 kHz.

After whole-cell recordings *in vivo* we obtained outside-out patches to ensure membrane re-sealing and processed the tissue for biocytin labeling. Mice were returned to deep isoflurane anesthesia, and perfused with PBS then 4% PFA in phosphate buffered saline (PBS).

Vibratome sections (60 μm) were cut coronally from whole brains embedded in agarose. Biocytin fills were labeled in free floating slices by overnight incubation at 4°C in PBS containing 0.01% Triton X-100 and Alexa594 conjugated streptavidin (1 μg/ml, Life Technologies), then washed thoroughly (12-24 h in PBS with 3 changes of solution) in the same wells, before being mounted on slides using Vectashield and imaged using a 10× objective on a Zeiss LSM510 confocal inverted microscope. All recordings included in the dataset were associated with labeled cells in the PVH, though for some recordings it was not possible to unambiguously match biocytin-labeled PVH neurons to the recorded cell due to extensive uptake of biocytin by multiple PVH neurons. For all recordings after shRNA *Scn9a* knock-down, biocytin labeling was observed in PVH neurons expressing the shRNA construct. However because of extensive biocytin uptake we could not unambiguously assign every recording to these neurons and thus analyzed all PVH neurons recorded from shRNA transduced mice as the *Scn9a* knock-down group.

Channelrhodopsin-assisted circuit mapping. Acute coronal slices were prepared from $Agrp^{Cre/+};Scn9a^{flox/flox}$ mice or $Agrp^{Cre/+};Scn9a^{wt/wt}$ mice (>7 weeks old) injected with *rAAV9.eF1a.dflox.ChR2(H134R)*. After a minimum of 2 weeks post-injection, whole-cell recordings were made from non-fluorescent cells in the arcuate nucleus, and IPSCs were recorded upon wide-field stimulation with 1 ms 488 nm light pulses delivered at 20 Hz.

Pharmacology. Recordings of EPSCs and intracellular voltage were done in the presence of Picrotoxin, except for the data shown in Figure S1F-G. Measurements of firing rate using cell-attached recordings were done without blockers. Drugs were bath applied with a Miniplus 3 (Gilson, FR) peristaltic pump perfusion system. Blockers and final concentrations were: Glutamate receptors (AP-5 50 μM, Tocris; Kynurenic Acid 2 mM, Sigma; NBQX 1 μM Tocris), $GABA_A$ receptors (Picrotoxin 50 μM, Sigma); Na_V channels (TTX 1μM, Tocris), K_V channels (TEA 10 mM, Sigma); Ca_V channels ($CdCl_2$ 100μM and $NiCl_2$ 50 μM; Sigma); $Na_v1.7$ (Protoxin-II

2 nM, Alomone Labs). Unless otherwise noted, slices were perfused in blockers at least 10 min prior to obtaining recordings, and all comparisons made are against the population of cells recorded in the absence of blockers.

Data analysis. Electrophysiology data was analyzed in Python 2.7 using custom written routines. The values for I_{NaP} reported in the text and in Figures 1 and 5 are for peak currents at -30 mV obtained with ramp protocols recorded in the presence of blockers of voltage-gated potassium and calcium channels (TEA, $CdCl_2$ and $NiCl_2$), and with the leak subtracted by fitting a linear function to the initial, linear part of the current response. In cells that passed the stability criteria, TTX was applied and the peak TTX-sensitive current directly measured in the same cell (e.g., Figure 1G), which was not significantly different from the above procedure. Membrane time constants were estimated for each cell by fitting a single exponential to the decay of a hyperpolarizing current step (-10 pA). EPSPs and IPSPs were detected automatically by threshold algorithm applied to the first derivative of the voltage traces. Values of EPSP decay times normalized to the membrane time constant were obtained by measuring the fractional decay from the EPSP at 100 ms after the EPSP onset, and dividing by the decay predicted by membrane time constant on a cell-by-cell basis. All membrane potential values reported have been corrected for junction potentials.

Supplemental References

Atasoy, D., Aponte, Y., Su, H.H., and Sternson, S.M. (2008). A FLEX switch targets Channelrhodopsin-2 to multiple cell types for imaging and long-range circuit mapping. J Neurosci *28*, 7025-7030.

Betley, J.N., Cao, Zhen Fang H., Ritola, Kimberly D., and Sternson, Scott M. (2013). Parallel, Redundant Circuit Organization for Homeostatic Control of Feeding Behavior. Cell *155*, 1337-1350.

Dow, L.E., Premsrirut, P.K., Zuber, J., Fellmann, C., McJunkin, K., Miething, C., Park, Y., Dickins, R.A., Hannon, G.J., and Lowe, S.W. (2012). A pipeline for the generation of shRNA transgenic mice. Nat Protoc *7*, 374-393.

Hempel, C.M., Sugino, K., and Nelson, S.B. (2007). A manual method for the purification of fluorescently labeled neurons from the mammalian brain. Nat Protoc *2*, 2924-2929.

Henry, F.E., Sugino, K., Tozer, A., Branco, T., and Sternson, S.M. (2015). Cell type-specific transcriptomics of hypothalamic energy-sensing neuron responses to weight-loss. Elife *4:e09800*.
Lee, D., Lin, B.J., and Lee, A.K. (2012). Hippocampal place fields emerge upon single-cell manipulation of excitability during behavior. Science *337*, 849-853.
Matveeva, O.V., Nazipova, N.N., Ogurtsov, A.Y., and Shabalina, S.A. (2012). Optimized models for design of efficient miR30-based shRNAs. Frontiers in genetics *3*, 163.
Stern, P., Astrof, S., Erkeland, S.J., Schustak, J., Sharp, P.A., and Hynes, R.O. (2008). A system for Cre-regulated RNA interference in vivo. Proc Natl Acad Sci U S A *105*, 13895-13900.
Weiss, J., Pyrski, M., Jacobi, E., Bufe, B., Willnecker, V., Schick, B., Zizzari, P., Gossage, S.J., Greer, C.A., Leinders-Zufall, T.*, et al.* (2011). Loss-of-function mutations in sodium channel Nav1.7 cause anosmia. Nature *472*, 186-190.
