# Supplementary material for: Near-Perfect Synaptic Integration by Nav1.7 in Hypothalamic Neurons Regulates Body Weight
Source: Cell. 2016 Jun 16;165(7):1749–61. doi: 10.1016/j.cell.2016.05.019 (PMC4912688; doi:10.1016/j.cell.2016.05.019)
Supplement: Document S2. Article plus Supplemental Information [file mmc2.pdf]

Cell

Near-Perfect Synaptic Integration by $Na_v1.7$ in Hypothalamic Neurons Regulates Body Weight

Graphical Abstract

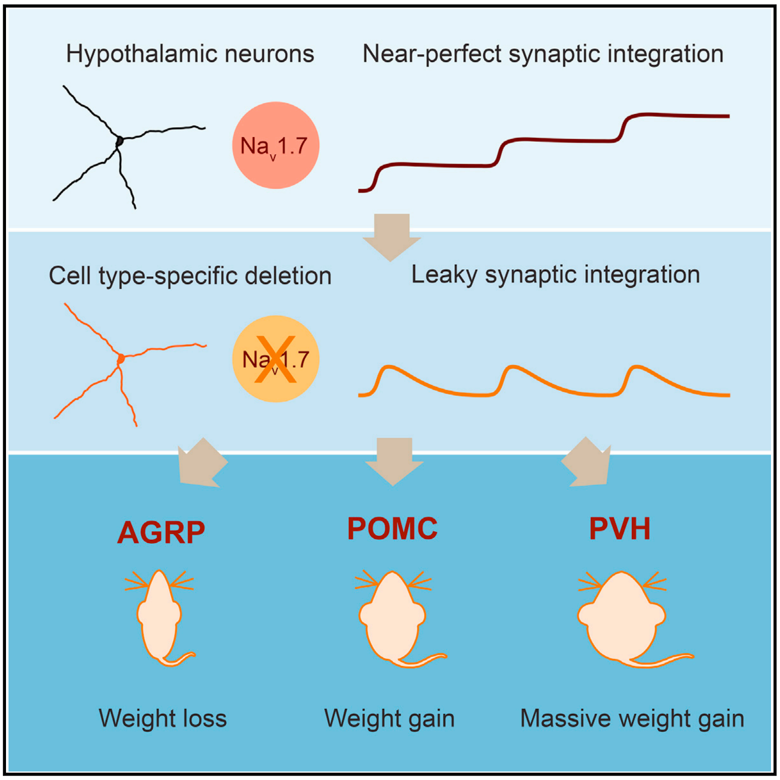

Highlights

- Hypothalamic neurons that regulate body weight are near-perfect synaptic integrators
- Near-perfect synaptic integration is observed in hypothalamic neurons in vivo
- Near-perfect synaptic integration depends on the voltage-gated sodium channel $Na_v1.7$
- Loss of $Na_v1.7$ in hypothalamic neurons disrupts regulation of body weight

Authors

Tiago Branco, Adam Tozer, Christopher J. Magnus, ..., Albert K. Lee, John N. Wood, Scott M. Sternson

Correspondence

tbranco@mrc-lmb.cam.ac.uk (T.B.),
sternsons@janelia.hhmi.org (S.M.S.)

In Brief

Neurons in the hypothalamus are capable of summing synaptic inputs received over long periods of time, providing insights into how long-term homeostatic functions like weight maintenance can be set by neural circuits.

Branco et al., 2016, Cell *165*, 1749–1761
June 16, 2016 © 2016 MRC Laboratory of Molecular Biology.
Published by Elsevier Inc.
http://dx.doi.org/10.1016/j.cell.2016.05.019

Near-Perfect Synaptic Integration by $Na_v1.7$ in Hypothalamic Neurons Regulates Body Weight

Tiago Branco,[1,2,*] Adam Tozer,[2] Christopher J. Magnus,[1] Ken Sugino,[1] Shinsuke Tanaka,[1] Albert K. Lee,[1] John N. Wood,[3] and Scott M. Sternson[1,*]
[1]Janelia Research Campus, Howard Hughes Medical Institute, 19700 Helix Drive, Ashburn, VA 20147, USA
[2]Division of Neurobiology, Medical Research Council Laboratory of Molecular Biology, Cambridge CB2 0QH, UK
[3]Molecular Nociception Group, Wolfson Institute for Biomedical Research, University College London, London WC1E 6BT, UK
*Correspondence: tbranco@mrc-lmb.cam.ac.uk (T.B.), sternsons@janelia.hhmi.org (S.M.S.)
http://dx.doi.org/10.1016/j.cell.2016.05.019

SUMMARY

Neurons are well suited for computations on millisecond timescales, but some neuronal circuits set behavioral states over long time periods, such as those involved in energy homeostasis. We found that multiple types of hypothalamic neurons, including those that oppositely regulate body weight, are specialized as near-perfect synaptic integrators that summate inputs over extended timescales. Excitatory postsynaptic potentials (EPSPs) are greatly prolonged, outlasting the neuronal membrane time-constant up to 10-fold. This is due to the voltage-gated sodium channel $Na_v1.7$ (*Scn9a*), previously associated with pain-sensation but not synaptic integration. *Scn9a* deletion in AGRP, POMC, or paraventricular hypothalamic neurons reduced EPSP duration, synaptic integration, and altered body weight in mice. In vivo whole-cell recordings in the hypothalamus confirmed near-perfect synaptic integration. These experiments show that integration of synaptic inputs over time by $Na_v1.7$ is critical for body weight regulation and reveal a mechanism for synaptic control of circuits regulating long term homeostatic functions.

INTRODUCTION

Synaptic integration mechanisms in neurons combine synaptic potentials to control action-potential firing. The manner in which neurons integrate synaptic inputs can vary extensively, and these processes underlie the different computations mediated by particular neuronal cell types. For example, cortical and hippocampal neurons, which process rapidly changing information, integrate thousands of inputs with rapid decay times (~10–50 ms) that often require precise temporal coincidence to elicit neuronal firing (Magee, 2000; Spruston, 2008). Synaptic integration properties are typically tied to the fundamental biophysical properties of all cellular membranes, which make neurons well-suited for responding on short timescales (Jack et al., 1975). However, neurons in many brain circuits operate over long timescales, despite having similar core membrane properties, and much less is known about how they integrate synaptic inputs.

AGRP neurons in the hypothalamic arcuate nucleus (ARC) regulate appetite and body weight on long timescales ranging from minutes to hours, in part, by integrating hormonal signals (Gao and Horvath, 2007), which includes hormone action on excitatory synaptic inputs (Yang et al., 2011). These neurons undergo marked synaptic plasticity in response to changing metabolic and hormonal states (Liu et al., 2012; Pinto et al., 2004; Yang et al., 2011), indicating an important role for synaptic control over AGRP neuron activity. Moreover, several sources of synaptic input have been identified (Krashes et al., 2014; Sternson et al., 2005; Wang et al., 2015) and found to be important for maintaining AGRP neuron activity. To understand the computational properties of AGRP neurons, we investigated their synaptic physiology in conjunction with neuronal modeling, cell type-specific RNA sequencing (RNA-seq) data, and gene expression perturbation methods to probe how excitatory synaptic inputs control AGRP neuron firing. We found remarkably efficient synaptic integration properties and determined an underlying molecular mechanism that is found in AGRP neurons and multiple other hypothalamic cell types, is observed in vivo, and is required for normal body weight regulation.

RESULTS

Efficient Synaptic Integration in AGRP Neurons

To examine the relationship of fast synaptic transmission to neuronal firing in energy homeostasis circuits, we started by measuring synaptic integration in AGRP neurons. In acute hypothalamic brain slices containing the ARC from adult (5–10 weeks) male *Npy*^{hrGFP} transgenic mice (arcuate NPY neurons co-express *Agrp*), we measured the input-output function of AGRP neurons using cell-attached recordings of the spontaneous spike rate and whole cell recordings of pharmacologically isolated spontaneous excitatory synaptic input (see Experimental Procedures). AGRP neurons received a mean excitatory input rate of 6.6 ± 0.8 Hz and fired action potentials at 2.6 ± 0.3 Hz (n = 16), which corresponds to 2.5:1 input-output conversion (Figure 1A). In the presence of the AMPA receptor antagonist NBQX (2,3-dihydroxy-6-nitro-7-sulfamoyl-benzo[f]quinoxaline-2,3-dione; 1 μM), spontaneous action potentials were not observed, showing that AGRP neuron firing requires integration

© 2016 MRC Laboratory of Molecular Biology. Published by Elsevier Inc.
This is an open access article under the CC BY license (http://creativecommons.org/licenses/by/4.0/).

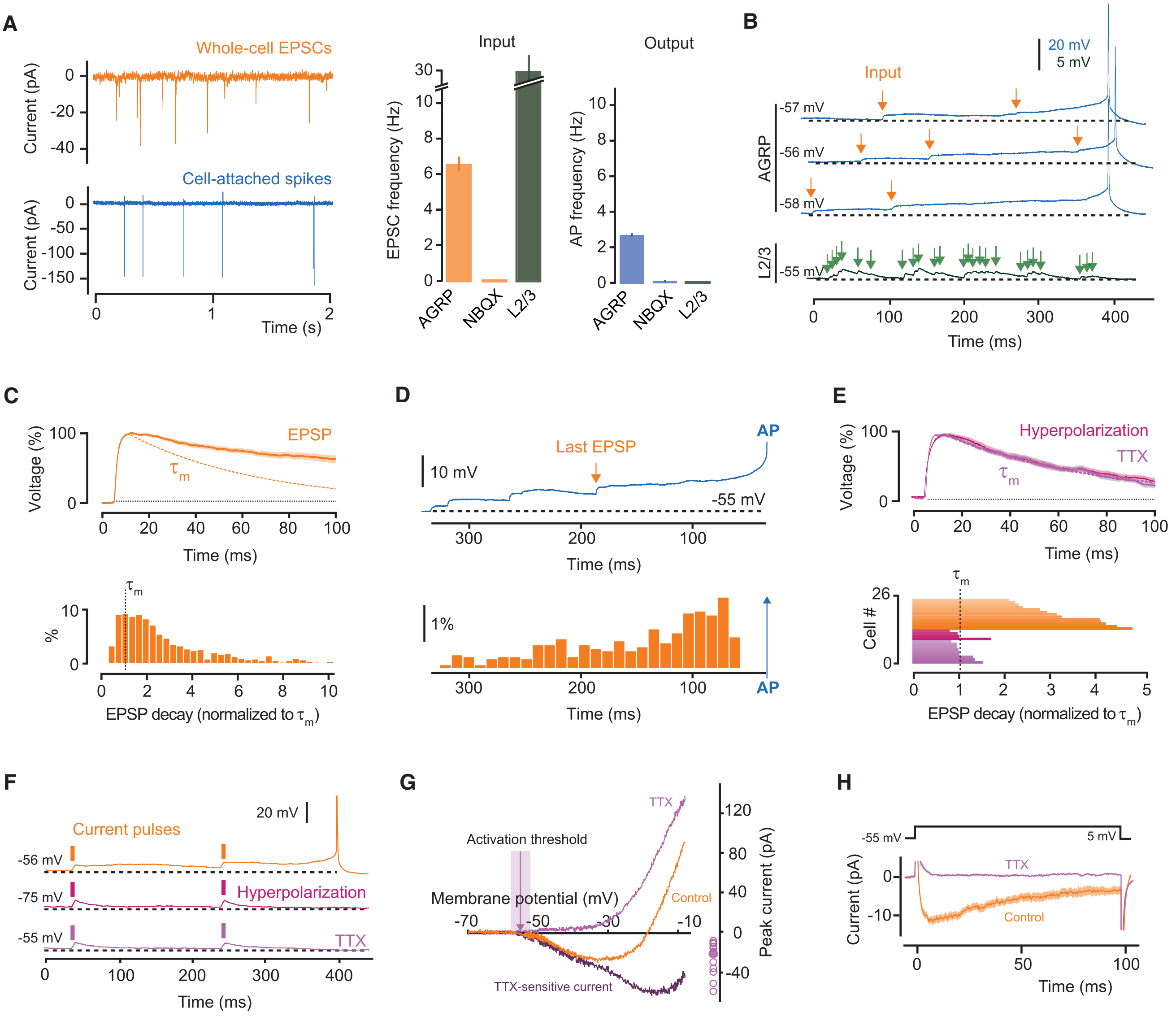

Figure 1. Persistent Sodium Current Prolongs EPSPs in AGRP Neurons

(A) Input-output relationship for AGRP neurons. Left: example traces. Right: summary data with mean frequency of spontaneous excitatory postsynaptic currents (EPSCs, input) and action potential currents (AP, output) for AGRP neurons in the absence and presence of the AMPA receptor antagonist, NBQX. Input-output relationship for cortical layer 2/3 (L2/3) pyramidal neurons is shown for comparison (green, n = 4).

(B) Voltage recording of spontaneous activity showing step-wise integration of excitatory synaptic input efficiently leading to action potential firing in AGRP neurons and failure to summate to threshold in cortical L2/3 neurons despite higher input frequency (bottom trace).

(C) Top: peak-scaled mean spontaneous EPSP for one cell shows decay much slower than the membrane time constant (τ_m, dashed orange line). Bottom: histogram for normalized EPSP decay times across all cells.

(D) Top: example trace illustrating the timing of the last EPSP before an action potential. Bottom: histogram of time of last EPSP preceding all action potentials (n = 9 cells).

(E) Top: somatic hyperpolarization and TTX shorten EPSP decay to the membrane time constant (purple dotted line). Bottom: mean EPSP decay time for each cell, including for untreated AGRP neurons (orange bars).

(F) Somatic injection of short current pulses (20 pA, 5 ms) reproduces step-wise integration (top trace), which was abolished by hyperpolarization or TTX.

(G) Isolation of a TTX-sensitive persistent current with slow voltage ramp (20 mV/s). Left: example traces from one cell. Right: average peak currents for each cell.

(H) Single depolarizing voltage step elicits a TTX-sensitive inward current (traces are averages for all cells). Data are represented as mean ± SEM. Lines with shaded areas are mean ± SEM.

See also Figure S1.

of synaptic input (Figure 1A). This remarkably efficient input-output function contrasts with input integration in other areas of the brain, where, for example, pyramidal cells in the cortex or hippocampus need an input rate on the order of 1 kHz (Branco and Häusser, 2011; Shadlen and Newsome, 1998) to produce a single action potential (see examples in Figures 1A and 1B).

Next, we examined biophysical and synaptic properties that underlie efficient integration of excitatory synaptic input in AGRP neurons. We used whole-cell voltage recordings and examined the membrane potential trajectory before spontaneous action potentials. Each action potential was preceded by exceptionally long-lasting EPSPs, which could persist up to 500 ms. This is remarkable because cellular membranes act as resistor-capacitor circuits, and synaptic potentials typically decay with the passive neuronal membrane time constant (τ_m, the product of cellular membrane electrical resistance and the membrane capacitance), but EPSPs in AGRP neurons decayed, on average, 3.3 ± 0.2 times more slowly than the neuronal membrane time constant (τ_m: 37.7 ± 4.0 ms, n = 13, paired t test, $p < 0.001$; Figures 1B, 1C, and S1A–S1E). Strikingly, individual EPSPs could last up to 10-fold longer than τ_m, in sharp contrast to the ~20 ms decay-times observed in cortical neurons (Iansek and Redman, 1973; Magee, 2000; Williams and Stuart, 2000). As a result of this unusually slow decay time course, EPSPs were integrated by AGRP neurons as stepwise increases in membrane potential (Figure 1D). Thus, on average, the last spontaneous EPSP occurred well before action potential onset (99.1 ± 5.8 ms, Figure 1D), and there were only 2.1 ± 0.1 synaptic events between the resting membrane potential and the action potential, which is in good agreement with the 2.5:1 input-output ratio (Figure 1A). Similar results were also observed for combined integration of excitatory and inhibitory input (Figures S1F and S1G). These experiments show that AGRP neurons are near-perfect synaptic integrators (Knight, 1972; Pressley and Troyer, 2009), whereby the voltage depolarization caused by each excitatory synaptic input can be sustained for considerably longer than the membrane time constant and can sum over long timescales. This mode of integration is fundamentally distinct from that used by neurons in brain regions where information processing relies on coincidence detection (König et al., 1996).

Persistent Sodium Current Is Required for Integration

The mismatch between the membrane time constant and EPSP duration in AGRP neurons suggests that synaptic responses are prolonged by an active postsynaptic conductance. To test this, we first hyperpolarized AGRP neurons by −20 mV (membrane potential: –77.5 ± 2.4 mV) to reduce activation of voltage-gated depolarizing conductances, and we found that EPSPs decayed much faster, so that their decay was no longer significantly different than the membrane time constant (110 ± 20% of τ_m; n = 4; paired t test, p = 0.52, Figure 1E). Responses to somatic injection of brief current pulses showed similar properties to spontaneous EPSPs, with long decay time constants and stepwise summation, that were also abolished with hyperpolarization (96% ± 4% of τ_m, n = 4, versus 233% ± 31% for control, n = 5; unpaired t test, p = 0.011; Figure 1F), and similar results were obtained with injections of mEPSC waveforms (Figure S1H), further confirming a role for active conductances in generating step-like changes in AGRP neuron membrane potential.

To identify the ion channel conductances required for prolonged EPSPs, we used pharmacology to block conductances previously associated with active input integration (Gulledge et al., 2005; Magee, 2000). Antagonists of NMDA receptors or L-type and T-type voltage-gated calcium channels did not significantly change input integration in AGRP neurons (Figures S1I–S1K). In contrast, blocking voltage-gated sodium channels with tetrodotoxin (TTX) abolished EPSP prolongation (111% ± 7% of τ_m; n = 9; paired t test, p = 0.13; Figure 1E), without changing the properties of spontaneous EPSCs (Figures S1L–S1N). Moreover, the decay time of responses to somatically injected current pulses was reduced by TTX to the membrane time-constant (94% ± 6% of τ_m for TTX, n = 5; paired t test, p = 0.12; Figure 1F), showing a critical role for voltage-gated sodium channels in synaptic integration in AGRP neurons.

Voltage-gated sodium channels can amplify synaptic potentials via a persistent sodium current (I_{NaP}), (Carter et al., 2012; Cummins et al., 1998; Farries et al., 2010; French et al., 1990; Fricker and Miles, 2000; Prescott and De Koninck, 2005; Stuart and Sakmann, 1995). I_{NaP} can be isolated using slow voltage-ramp protocols, which inactivate the much larger fast transient sodium channel conductances while activating I_{NaP}. To test whether I_{NaP} is present in AGRP neurons, we used this protocol while blocking other voltage-gated channels, and we observed a TTX-sensitive persistent current (activation threshold: −56.4 ± 2.3 mV, average current at −30 mV: −23.3 ± 3.2 pA, n = 13, Figure 1G), which was 0.95% of the transient inward current (peak amplitude: −2.4 ± 0.3 nA). Even with potassium conductances intact, the net current elicited by small (+5 mV) voltage steps from the resting potential was inward (−5.5 ± 0.7 pA at 50 ms after onset, n = 15) and TTX-sensitive (Figure 1H), indicating that I_{NaP} can be activated by the small depolarization produced by synaptic input and thus support EPSP prolongation in AGRP neurons.

These experiments show that the intrinsic conductances present in AGRP neurons favor activation of sustained depolarizing currents close to the resting membrane potential. As the net persistent current only becomes outward near the action potential threshold, these properties are well-suited to link synaptic input to action potential initiation, with each excitatory synaptic input activating I_{NaP}, and moving the membrane potential toward the action potential threshold in a stepwise fashion. To further understand the underlying biophysical mechanisms, we also measured the total potassium channel conductance (Figure 2A) and developed computational models for AGRP neurons. We first defined the minimum biophysical conditions sufficient to generate prolonged synaptic potentials in a single compartment model equipped with I_{NaP}, voltage-gated potassium channels, and passive properties that matched our experimentally measured voltage-clamp data (Figure 2B) from AGRP neurons. This model reproduced long-lasting EPSPs, similar to those that we observed experimentally in AGRP neurons. We also systematically varied the model parameters and found that EPSP prolongation occurs in a narrow band of I_{NaP} and voltage-gated potassium conductances (Figure 2C). This is required so that I_{NaP} activation generates net inward current, but does not lead to

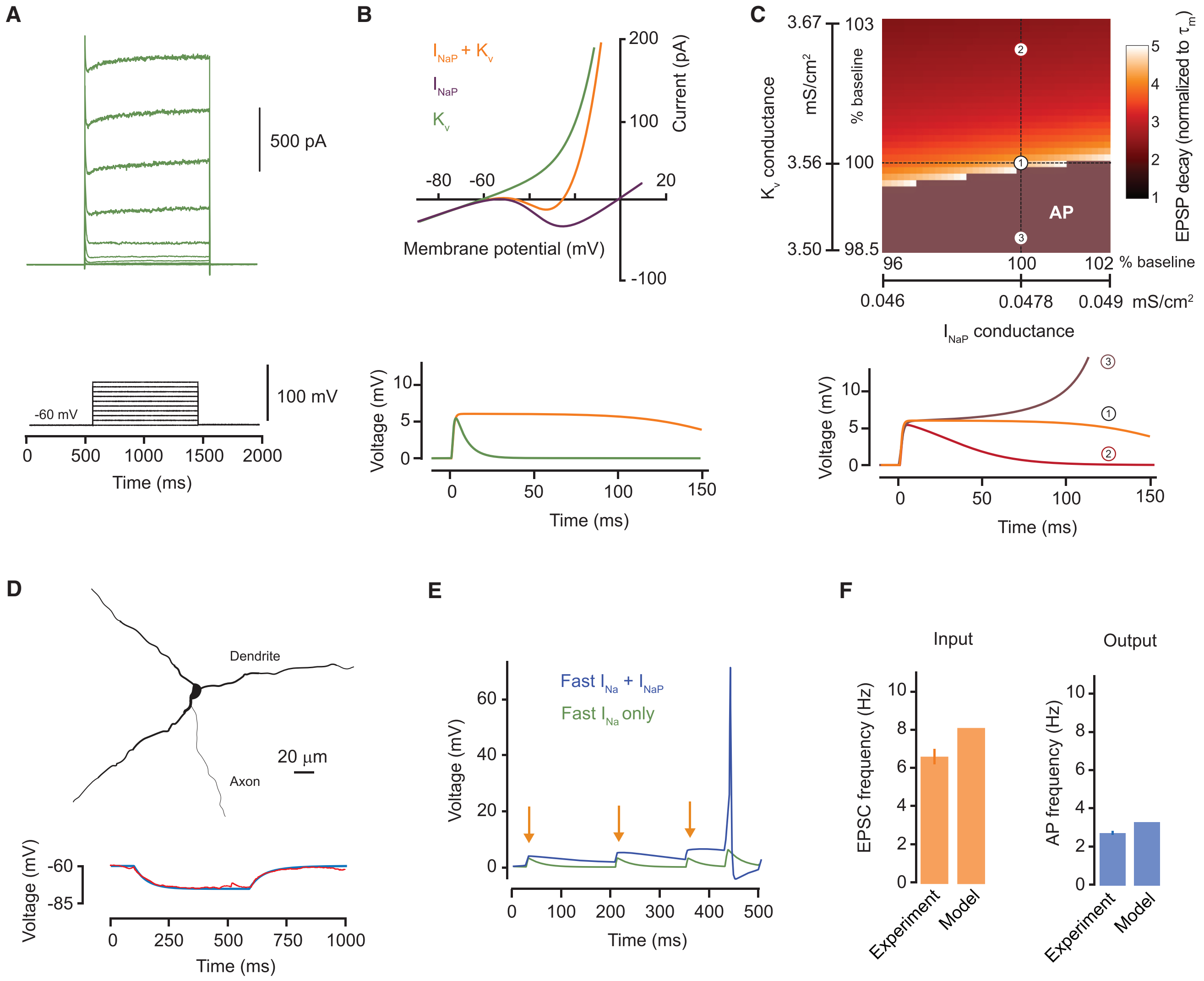

Figure 2. Compartmental Model of Input Integration in AGRP Neurons

(A) Experimentally measured potassium currents from AGRP neurons in response to voltage steps (example traces from one cell) used to constrain the model.

(B) Top: current responses to slow voltage ramps in a single compartment model with a leak conductance and either I_{NaP}, voltage-gated potassium channels (K_v) or both, show currents densities matching those recorded in AGRP neurons. Bottom: calculated membrane potential response to a single synaptic input shows that I_{NaP} is sufficient to reproduce long-lasting EPSPs.

(C) Top: varying I_{NaP} and K_v conductance densities in the single compartment model by ~5% disrupts EPSP prolongation, with high K_v/I_{NaP} ratios producing fast decaying EPSPs and low K_v/I_{NaP} ratios leading to action potentials (AP), showing a critical synergy between I_{NaP} and voltage-gated potassium channels. Bottom: example EPSP traces for baseline (1) and two different conductance ratios.

(D) Morphology of a reconstructed AGRP neuron used to produce a multi-compartmental model (top, axon partially shown) and the calculated membrane potential response to a 10 pA current step (bottom, red trace is experimental data, blue is simulated data).

(E) The multi-compartmental model replicates the membrane potential response to step-wise integration of excitatory synaptic input in the presence of I_{NaP}.

(F) Excitatory synaptic input with Poisson statistics and a mean rate of 8 Hz is efficiently integrated into action potentials and matches the experimentally measured input-output relationship of AGRP neurons. Data are represented as mean ± SEM.

See also Figure S2.

runaway depolarization and achieves relatively stable voltage levels after synaptic input (Figures S2A and S2B). Additional key factors for generating step-like EPSPs in AGRP neurons are their high input resistance, which converts small amounts of I_{NaP} into large enough depolarization to support current regeneration, and a resting potential close to the I_{NaP} activation threshold (Figures S2C and S2D). We next developed a multi-compartmental model of AGRP neurons based on an experimentally reconstructed cell, which also reproduced step-wise integration and efficient input-output function (Figure 2D–2F). Thus, the combination of I_{NaP}, favorably tuned potassium channels, a relatively depolarized resting membrane potential, and high input resistance support near-perfect synaptic integration in AGRP neurons.

Input Integration Is Associated with Na_v1.7

Voltage-gated sodium channels are composed of a variety of alpha subunits with different biophysical properties (Catterall, 2000). Although persistent sodium currents can, in principle, be generated by any voltage-gated sodium channel (Taddese and Bean, 2002), we aimed to identify the molecular basis of I_{NaP} in AGRP neurons. Analysis of RNA sequencing data from AGRP neurons (Henry et al., 2015) showed several common neuronally expressed voltage-gated sodium channels (*Scn1a-3a*, Figure 3A) as well as unexpected expression of *Scn9a*, which encodes Na_v1.7. Na_v1.7 has been previously described in pain-sensing neurons and olfactory sensory neurons (Cox et al., 2006; Dib-Hajj et al., 2013; Weiss et al., 2011), where it has been suggested to generate a persistent sodium current that helps to bring subthreshold membrane fluctuations associated with nociception to the action potential firing threshold or to mediate action potential propagation and synaptic release in olfactory sensory neurons (Cummins et al., 1998; Weiss et al., 2011). Although Na_v1.7 has not been examined elsewhere in the brain, we tested the contribution of Na_v1.7 to I_{NaP} and EPSP prolongation in AGRP neurons by using Protoxin-II, a sodium channel blocker that has a 100-fold higher affinity for Na_v1.7 over other voltage-gated sodium channels (Schmalhofer et al., 2008). Protoxin-II significantly reduced the current amplitude evoked by a small voltage step (42.6% ± 19.8% of I_{NaP} in absence of Prototoxin-II, n = 12; unpaired t test, $p < 0.05$), and also decreased the decay time of EPSPs (42.3% ± 4% of decay time in absence of Prototoxin-II, n = 8; unpaired t test, $p < 0.001$), indicating that Na_v1.7 might contribute to synaptic integration in AGRP neurons (Figures 3B and 3C).

Next, we investigated *Scn9a* expression by RNA fluorescent in situ hybridization (FISH). *Scn9a* was highly expressed in the ARC, the dorsal medial hypothalamic nucleus (DMH), and the paraventricular hypothalamic nucleus (PVH); but not in the ventromedial hypothalamic nucleus (VMH), medial amygdala (MeA), ventral hippocampus dentate gyrus (vDG), or reticular thalamus (RT) (Figures 3D–3F). Interestingly, neurons in the PVH, which express *Scn9a*, showed prolonged EPSPs (335% ± 39% of τ_m, n = 10 paired t test, p = 0.002), whereas VMH neurons have low expression of *Scn9a* and do not have prolonged EPSPs (130% ± 12% of τ_m, n = 6; paired t test, p = 0.10). Thus, this synaptic integration property tracks the relative expression levels of *Scn9a* in at least three hypothalamic regions (Figure 3G). Double-label RNA-FISH in the ARC revealed 99.7% co-localization of *Scn9a* with *Agrp* transcripts (327/328 AGRP neurons; Figure 3H). Moreover, *Scn9a* expression in the ARC was not restricted to AGRP neurons. We also found that 99.4% of POMC neurons, an intermingled ARC neuron population with opposing effects on energy homeostasis, expressed *Scn9a* (168/169 POMC neurons; Figure 3I). Based on the correspondence between *Scn9a* expression in AGRP neurons and prolonged EPSP kinetics, we expected that POMC neurons might have synaptic integration properties that are similar to AGRP neurons. This prediction was confirmed by whole-cell recordings from POMC neurons in *Pomc*^{topazFP} transgenic mice, which showed sustained EPSPs preceding action potentials, as well as a TTX-sensitive net inward current in response to step-depolarization from the resting membrane potential (Figures 3J–3L). Therefore, *Scn9a* expression is associated with sustained EPSPs and near-perfect synaptic integration properties in several hypothalamic cell types tested here, including AGRP, POMC, and PVH neurons, which are critical regulators of energy homeostasis.

Efficient Input Integration Requires *Scn9a*

To confirm the molecular basis of I_{NaP} in AGRP neurons we first developed a recombinant adeno-associated viral (rAAV) vector for Cre recombinase-dependent, cell-type-specific RNA-interference ("knock-down") of *Scn9a* in the brain. This method couples reporter gene expression (humanized *Renilla* green fluorescent protein [hrGFP]) to RNA interference with a microRNA (*miR30*) cassette that was modified (Stegmeier et al., 2005; Stern et al., 2008) to encode a shRNA sequence for *Scn9a* in the 3′-untranslated region, allowing identification of neurons transduced with the short hairpin RNA (shRNA) (Figures 4A and 4B). This shRNA strongly reduced the amplitude of both transient and persistent sodium currents in HEK cells stably expressing murine Na_v1.7, while these currents were not substantially changed with a scrambled shRNA vector (Figures 4C–4G). Cre-conditional expression of rAAV2/9-*CAG::FLEX-rev-hrGFP:mir30(Scn9a)* in *Agrp*^{Cre} mice (AGRP$^{sh(Scn9a)}$ mice, Figures 4H and 4I) reduced EPSP duration resulting in synaptic potentials that decayed with the membrane time constant (AGRP$^{sh(Scn9a)}$: 116% ± 8% of τ_m, n = 14; *Npy*^{hrGFP}: 330% ± 20% of τ_m, n = 13; unpaired t test, $p < 0.001$), whereas expression of a scrambled *Scn9a* shRNA sequence maintained prolonged EPSPs (AGRP$^{sh(Scn9a\text{-}scram)}$: 271% ± 3% of τ_m, n = 7; *Npy*^{hrGFP}: 330% ± 20% of τ_m, n = 13; unpaired t test, p = 0.15, Figure 5A). Voltage-clamp ramps and voltage-step protocols showed marked reduction of I_{NaP} from AGRP$^{sh(Scn9a)}$ mice compared to scrambled *Scn9a* shRNA-expressing control cells (71.4% ± 2.6% reduction for ramps, n = 11 AGRP$^{sh(Scn9a)}$, n = 7 AGRP$^{sh(Scn9a\text{-}scram)}$; U test, $p < 0.001$, 85.4% ± 5.8% reduction for steps, n = 9 AGRP$^{sh(Scn9a)}$, n = 4 AGRP$^{sh(Scn9a\text{-}scram)}$; U test, p = 0.003; Figure 5B), consistent with a key role of the Na_v1.7 conductance for sustaining EPSPs. Importantly, *Scn9a* knock-down did not significantly affect input resistance (90.1% ± 8% of scrambled, unpaired t test, p = 0.56) or the membrane time constant (98% ± 13% of scrambled, unpaired t test, p = 0.92), suggesting that the more rapid EPSP decay was specifically due to the reduction of Na_v1.7-dependent I_{NaP} (Figure S3). Moreover, the voltage threshold for action potential firing was not significantly changed by *Scn9a* knock-down (Figure S3), and accordingly, the peak transient inward current was not significantly affected by *Scn9a* knock-down (AGRP$^{sh(Scn9a)}$: −2.4 ± 0.2 nA, n = 12; AGRP$^{sh(Scn9a\text{-}scram)}$: −2.6 ± 0.3 nA, n = 6; unpaired t test, p = 0.50). This is consistent with the endogenous expression of other voltage-gated sodium channels (Figure 3A), which may be upregulated to compensate for the loss of Na_v1.7-dependent transient current as has been observed in neurons after ablation of other sodium channels (Van Wart and Matthews, 2006; Vega et al., 2008). As expected from the loss of a persistent inward current, the rheobase was shifted to the right (52.8% ± 13.2% reduction in firing rate for 20 pA steps, n = 10 AGRP$^{sh(Scn9a)}$, n = 7 AGRP$^{sh(Scn9a\text{-}scram)}$; unpaired t test,

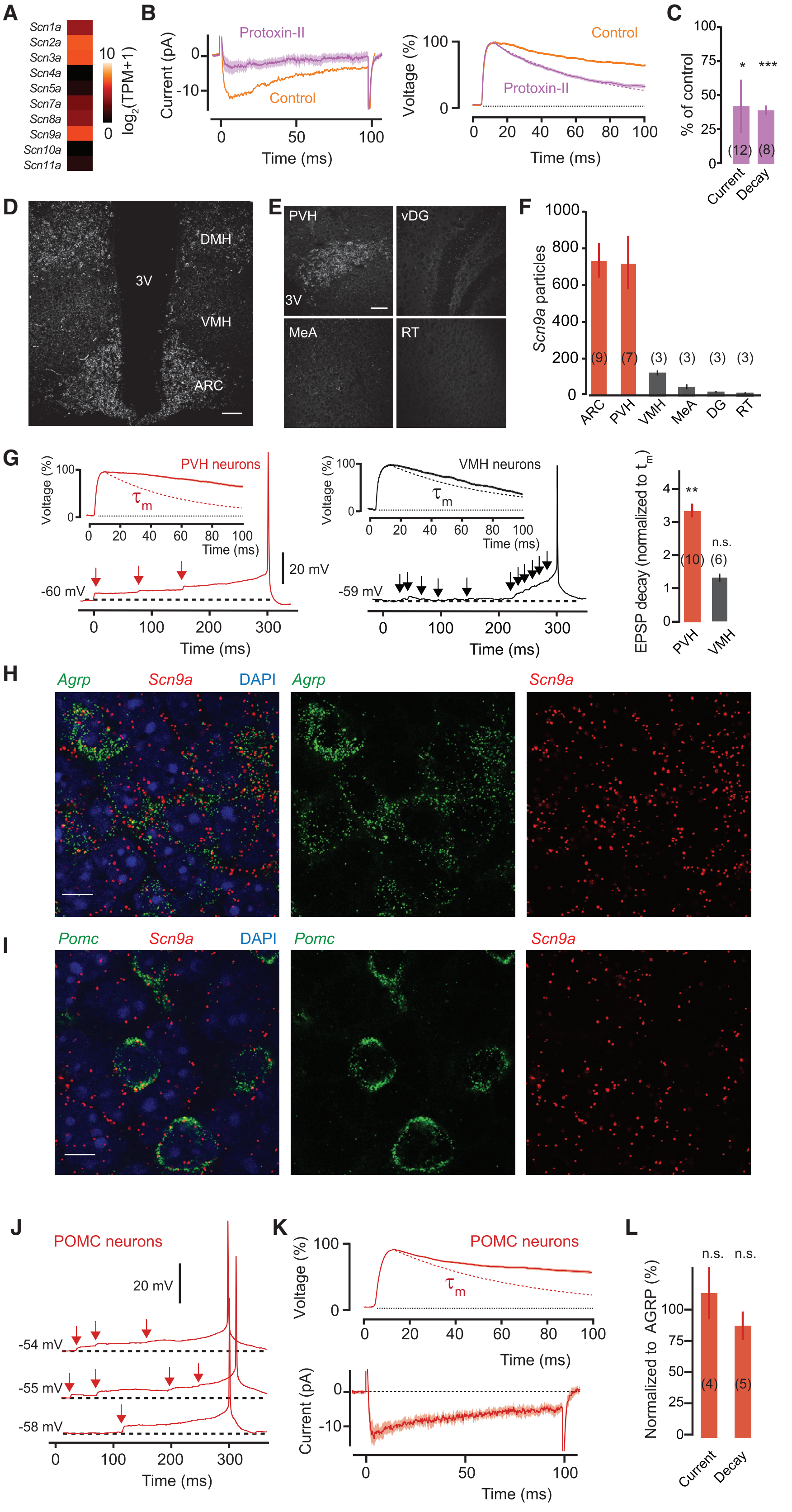

Figure 3. $Na_v1.7$ Is Expressed in AGRP and POMC Neurons

(A) Mean RNA expression levels of sodium channel alpha subunits from RNA sequencing of AGRP neurons (n = 5 samples, each from 1 mouse). *Scn9a*: $Na_v1.7$. TPM, transcripts per million.

(B) The $Na_v1.7$ selective blocker Protoxin-II reduces the net inward current (left) and shortens EPSP decay (right, τ_m, purple dashed line) across all cells (n = 8) relative to untreated AGRP neurons.

(C) Summary data for I_{NaP} and EPSP decay with Protoxin-II normalized to values from untreated AGRP neurons. Sample sizes in parentheses.

(D) RNA-fluorescent in situ hybridization (FISH) from *Scn9a* (white) shows strong labeling in the ARC and DMH, but not in the VMH. 3V, third ventricle. Scale, 100 μm.

(E) RNA-FISH for *Scn9a* in the PVH, ventral hippocampus dentate gyrus (vDG), medial amygdala (MeA), and reticular thalamus (RT). Scale, 30 μm.

(F) Mean *Scn9a* labeling in several brain regions. Sample sizes in parentheses from two mice.

(G) PVH neurons (left, n = 10) also show efficient synaptic integration and prolonged EPSPs, but VMH neurons (middle, n = 6) require coincident input to fire. τ_m, dashed line. Right: summary data for EPSP decay times (paired t tests versus τ_m).

(H and I) Double RNA-FISH for *Agrp* (green) and *Scn9a* (red) (H) or *Pomc* (green) and *Scn9a* (I) shows extensive colocalization. Blue, DAPI. Scale, 10 μm.

(J) POMC neurons show stepwise integration of excitatory input.

(K) Top: prolonged EPSPs (τ_m: red dashed line, n = 5). Bottom: net inward currents in response to 5 mV step depolarization (n = 4).

(L) Net inward current and EPSP decay time are similar to AGRP neurons (unpaired t test versus *Npy*^{hrGFP} cells, p = 0.66 for current and p = 0.39 for decay). Sample sizes in parentheses. Bar graphs or lines with shaded areas show mean ± SEM. ***$p < 0.001$, *$p < 0.05$, n.s. $p > 0.05$.

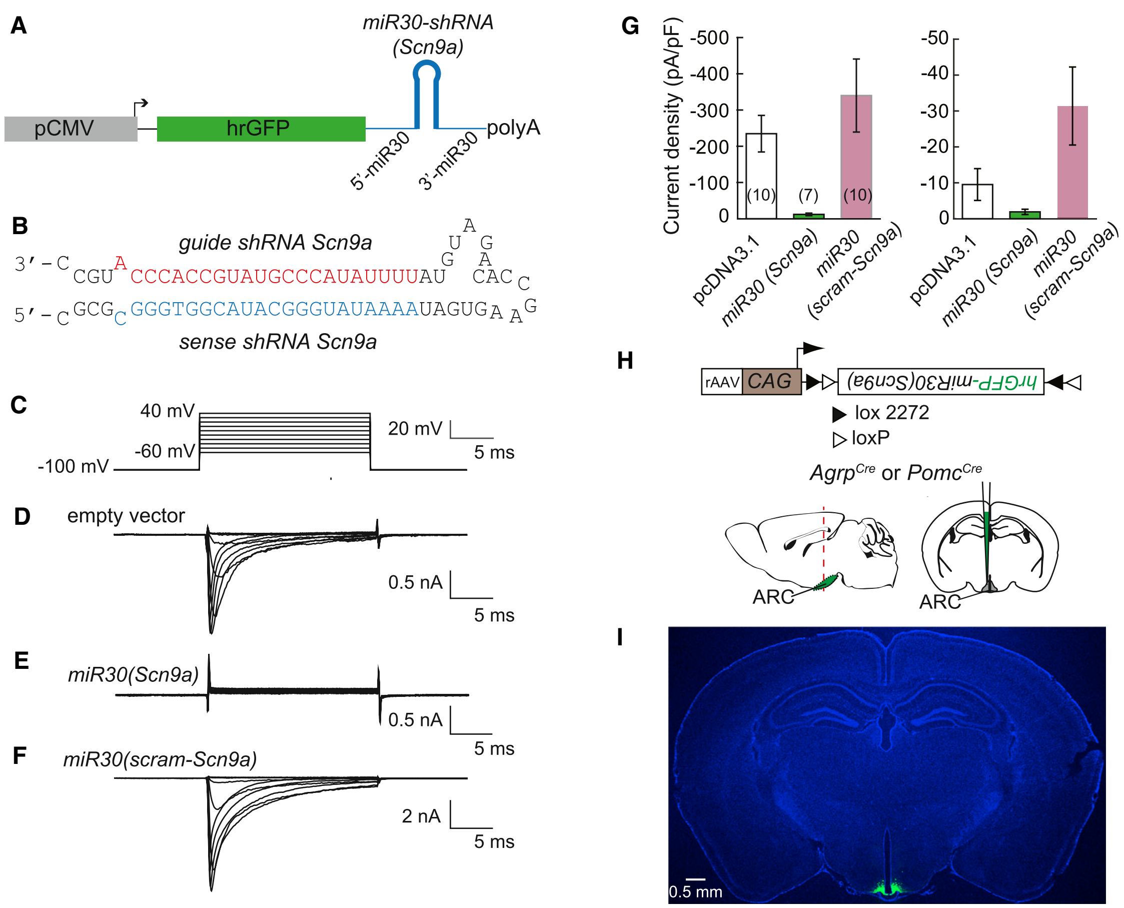

Figure 4. Cell-Type-Specific Tandem Fluorescent Protein/*miR30*-Based *Scn9a* Knockdown

(A) Construct design for validation of tandem fluorescent protein/*miR30*-based *Scn9a* knockdown in cultured cells. pCMV, cytomegalovirus promoter and enhancer; polyA, polyadenylation sequence.

(B) Sequence for shRNA for *Scn9a* knockdown.

(C–F) Voltage-gated currents in response to voltage steps from HEK cells stably expressing $Na_v1.7$ (C) after transfection with empty pcDNA3.1 vector (D), *miR30(Scn9a)*-containing vector (E), or *miR30(scrambled-Scn9a)*-containing vector (F).

(G) Effect of knockdown constructs in HEK cells stably expressing $Na_v1.7$. Left: peak current density in response to voltage steps (Kruskal-Wallis test, $p < 0.001$). Right: persistent current density in response to voltage step to −10 mV (Kruskal-Wallis test, $p = 0.005$). Sample sizes in parentheses. Data are represented as mean ± SEM.

(H) Construct design of Cre-dependent rAAV vector (top) for cell-type-selective targeting to AGRP or POMC neurons in *Agrp*^{Cre} or *Pomc*^{Cre} mice, respectively, with brain diagrams shown in sagittal and coronal cross sections.

(I) Image of coronal brain section from *Agrp*^{Cre} mouse bilaterally expressing *hrGFP-miR30(Scn9a)* in the ARC after Cre-dependent virus transduction.

$p < 0.05$), but the peak firing rate was not significantly different (AGRP$^{sh(Scn9a)}$: 28.6 ± 4.0 Hz, AGRP$^{sh(Scn9a\text{-}scram)}$: 33.5 ± 4.6 Hz; unpaired t test, $p = 0.89$; Figure 5C). *Scn9a* knock-down also greatly increased the number of spontaneous EPSPs required to reach action potential threshold (Figure 5D). Consistent with this, the input-output function was severely disrupted by *Scn9a* knock-down, which resulted in a 39:1 transformation ratio, corresponding to a 1,220% change, reflecting the failure of most inputs to contribute to action potential firing (firing rate for AGRP$^{sh(Scn9a)}$: 0.18 ± 0.1 Hz; n = 6; AGRP$^{sh(Scn9a\text{-}scram)}$: 2.5 ± 0.3 Hz, n = 5; U test, $p = 0.004$; Figure 5E). These results show that I_{NaP} via $Na_v1.7$ is critical for near-perfect integration in AGRP neurons. In agreement with *Scn9a* serving a similar role in POMC neurons, Cre-conditional *Scn9a* knock-down in *Pomc*^{Cre} mice produced a corresponding change in EPSP properties, strongly reducing EPSP decay time to the membrane time constant (decay = 109.7 ± 8% of τ_m, n = 4, paired t test, $p = 0.36$) and eliminating I_{NaP} (3.4 ± 2.5% of control *Pomc*^{topazFP} cells, n = 4, unpaired t test, $p < 0.01$) (Figure 5F). *Scn9a* knock-down in POMC neurons also greatly disrupted the input-output function (POMC$^{sh(Scn9a)}$: 65.6 ± 4.5 inputs/AP; *Pomc*^{topazFP}: 3.2 ± 0.5 inputs/AP; unpaired t test, $p < 0.001$; Figure 5G). Taken together, these experiments show that prolonged EPSPs in AGRP and POMC neurons are dependent on I_{NaP} mediated by $Na_v1.7$. $Na_v1.7$ was previously thought to be engaged primarily in nociception and olfaction, but here we show this channel to be widely expressed in the hypothalamus where it is essential for efficient synaptic integration.

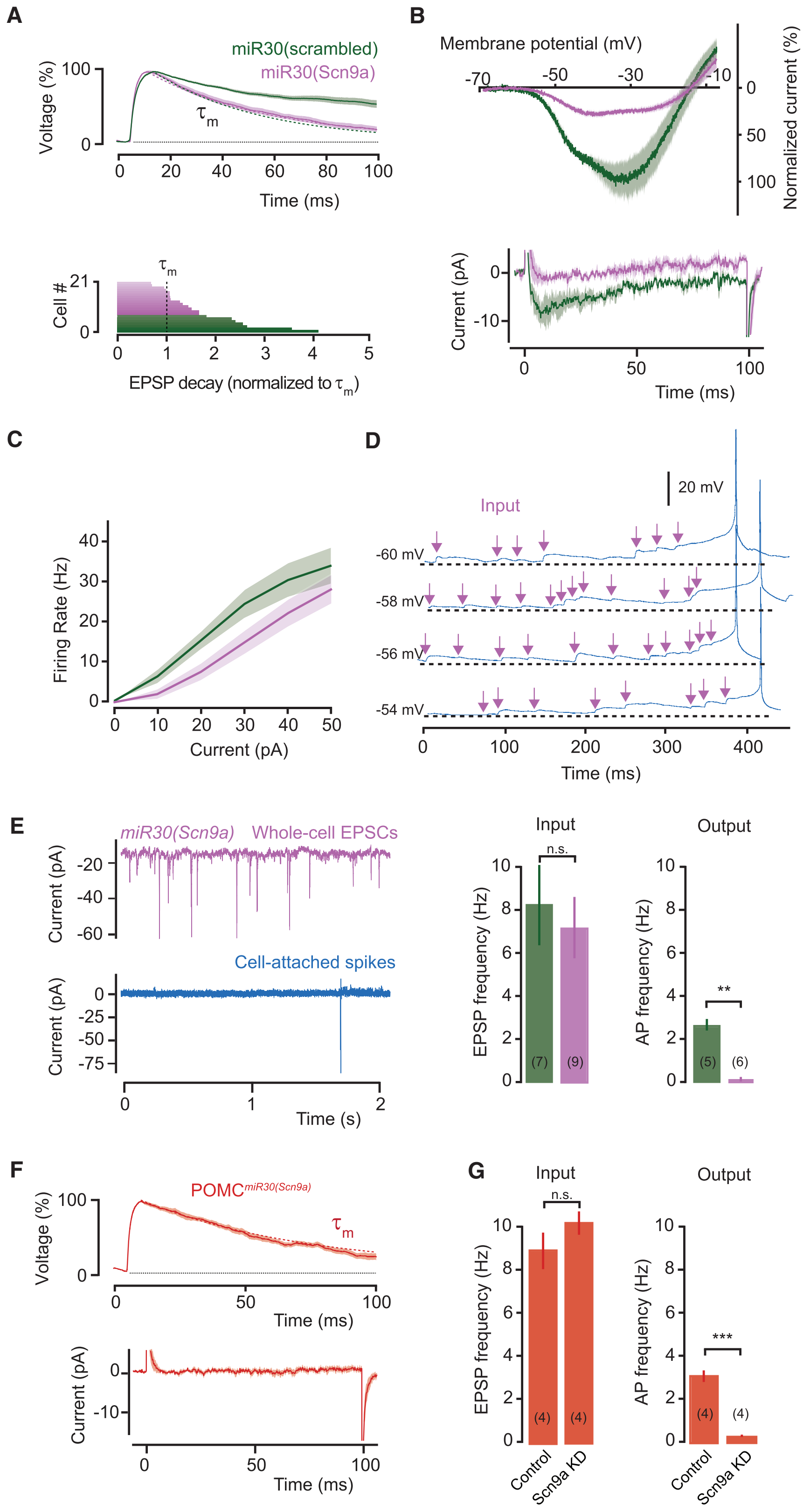

Figure 5. Efficient Synaptic Integration in AGRP and POMC Neurons Requires *Scn9a*

(A) Knockdown of *Scn9a* with *hrGFP-miR30(Scn9a)* abolishes EPSP prolongation in AGRP neurons, while *hrGFP-miR30(scrambled-Scn9a)* did not (top, example cells, purple and green dashed lines: τ_m). Bottom: mean EPSP decays normalized to τ_m for each cell.

(B) Persistent sodium current in slow ramp voltage-clamp protocols for *miR30(Scn9a)* and *miR30(scrambled-Scn9a)* in AGRP neurons (top, mean ± SEM across cells), and net current to a depolarizing step (bottom, mean ± SEM across cells).

(C) *Scn9a* knockdown increases the rheobase in AGRP neurons.

(D) Synaptic integration in AGRP neurons is severely disrupted after *Scn9a* knockdown, corresponding to an increase in the number of spontaneous EPSPs before an action potential.

(E) Input-output function of *miR30(Scn9a)*-expressing AGRP neurons shows almost no spontaneous action potentials despite normal input rates (left, example traces; right, summary data).

(F) *Scn9a* knockdown in POMC neurons abolishes EPSP prolongation (top, τ_m, dashed line) and persistent current (bottom).

(G) *Scn9a* knockdown in POMC neurons also disrupts the input-output function. Samples sizes in parentheses. Bar graphs or lines with shaded areas show mean ± SEM. ***$p < 0.001$, **$p < 0.01$, n.s. $p > 0.05$.

See also Figure S3.

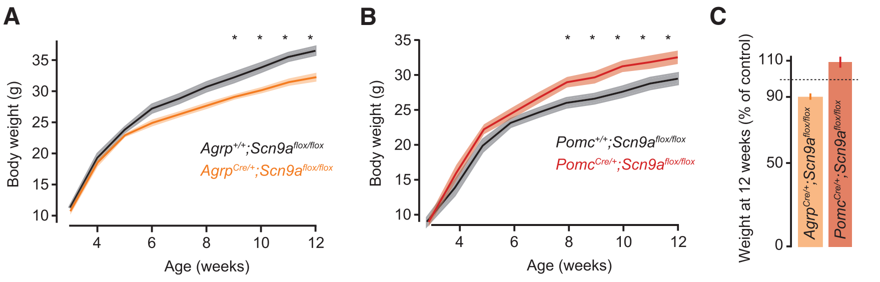

Figure 6. *Scn9a* in AGRP and POMC Neurons Is Required to Maintain Body Weight
(A and B) Weekly body weight for (A) *Agrp*$^{Cre/+}$;*Scn9a*$^{flox/flox}$ (n = 8) or (B) *Pomc*$^{Cre/+}$;*Scn9a*$^{flox/flox}$ mice (n = 12) and Cre-negative litter-mates (n = 11 and n = 16, respectively). Holm-Sidak correction for multiple comparisons.
(C) Body weights normalized to Cre-negative littermate controls. Bar graphs or lines with shaded areas show mean ± SEM. *p < 0.05.
See also Figure S4.

Scn9a Is Required for Body Weight Regulation

AGRP and POMC neurons play an important role in energy homeostasis. To test the necessity of Na$_V$1.7-dependent synaptic integration for control of body weight, we conditionally deleted *Scn9a* selectively in AGRP or POMC neurons using *Agrp*$^{Cre/+}$;*Scn9a*$^{flox/flox}$ or *Pomc*$^{Cre/+}$;*Scn9a*$^{flox/flox}$ mice, which produced a similar electrophysiological profile to *Scn9a* knock-down (Figures S4A–S4D). Although *Scn9a*$^{-/-}$ mice are reported to lose synaptic transmission in olfactory sensory neurons (Weiss et al., 2011), *Agrp*$^{Cre/+}$;*Scn9a*$^{flox/flox}$ mice maintained synaptic transmission to downstream neurons (Figures S4E and S4F). Thus, loss of *Scn9a* in these neurons alters synaptic input integration but not their capability to produce synaptic output.

Mice with cell-type-specific loss of *Scn9a* that were fed on a regular mouse diet developed significant body weight differences after 8 weeks. *Agrp*$^{Cre/+}$;*Scn9a*$^{flox/flox}$ mice showed reduced body weight relative to Cre-negative littermate controls (−8.6% ± 1.9% at 12 weeks, two-way ANOVA with repeated-measures, genotype: $F_{1,234} = 5.4$, p = 0.029; time: $F_{9,234} = 741$, p < 0.001; interaction: $F_{9,234} = 4.2$, p < 0.001; Figures 6A and 6C). Conversely, *Pomc*$^{Cre/+}$;*Scn9a*$^{flox/flox}$ mice increased body weight relative to Cre-negative littermate controls (+11% ± 2.7% at 12 weeks, two-way ANOVA with repeated-measures, genotype: $F_{1,180} = 3.8$, p = 0.066; time: $F_{9,180} = 527$, p < 0.001; interaction: $F_{9,180} = 5.7$, p = 0.015; Figures 6B and 6C), consistent with the role of these two cell populations in energy homeostasis. Therefore, in AGRP and POMC neurons, Na$_V$1.7 is essential for efficient synaptic integration as well as the opposite functions of these two homeostatic neuron populations for body weight regulation.

Near-Perfect Synaptic Integration In Vivo

To determine whether the step-wise synaptic integration supported by Na$_V$1.7 is observed in vivo, we performed whole-cell patch clamp recordings from hypothalamic neurons in anesthetized mice. For this, we targeted neurons in the PVH because they show pronounced EPSP prolongation ex vivo (Figures 3E–3G) that requires *Scn9a* expression (Figure S5), and, unlike neurons in the arcuate nucleus, we found them to be accessible to whole-cell recordings in vivo. Recording location was verified by biocytin labeling (Figure 7A; Supplemental Experimental Procedures).

PVH neurons fired spontaneously at 2.5 ± 0.9 Hz from a resting membrane potential of −51.3 ± 2 mV and showed similar biophysical properties to neurons ex vivo (n = 5; Figure 7B); although input resistance was lower (input resistance = 0.52 ± 0.05 GOhm in vivo versus 1.24 ± 0.07 GOhm ex vivo; τ_m, = 44 ± 5.6 ms in vivo versus 33.4 ± 3.3 ms ex vivo). Recordings of spontaneous activity showed a low rate of excitatory input (15.3 ± 1.4 Hz), as well as synaptic inhibition (9.9 ± 1.3 Hz). Similar to ex vivo recordings, PVH cells in vivo showed APs preceded by prolonged EPSPs that summated in a step-wise fashion, despite the presence of synaptic inhibition (Figure 7C). Excitatory synaptic potentials in vivo decayed 3.95 ± 0.3 times slower than the membrane time constant (paired t test, p = 0.0029) and EPSP prolongation was abolished by somatic hyperpolarization (120% ± 10% of τ_m; n = 3; paired t test, p = 0.24; Figures 7D and 7H). To further confirm the role of *Scn9a* in synaptic integration in vivo we performed shRNA *Scn9a* knock-down in PVH neurons. In recordings from shRNA transduced mice, EPSP decay in vivo was reduced to the membrane time constant (125% ± 22% of τ_m, n = 5; paired t test, p = 0.37) and step-wise synaptic integration was abolished (Figures 7E–7H). As a consequence, the resting firing rate of PVH$^{sh(Scn9a)}$ neurons in vivo was reduced >10-fold (Figure 7I; 0.18 ± 0.03 Hz, U test, p = 0.014) despite input rates similar to wild-type mice (EPSPs: 17.4 ± 1.4 Hz, t test, p = 0.40; IPSPs: 9.8 ± 0.9 Hz, t test, p = 0.94). Because of the established role of PVH neurons in energy homeostasis (Tolson et al., 2010), we also tested the necessity of *Scn9a*-dependent synaptic integration in body weight control by virally targeting Cre-EGFP to the PVH of *Scn9a*$^{flox/flox}$ mice, which eliminated efficient input integration but not the ability of PVH neurons to fire action potentials (Figure S5). Loss of *Scn9a* in the PVH dramatically increased in body weight (Figures 7J and 7K). After only 4 weeks, *PVH*$^{Cre/+}$;*Scn9a*$^{flox/flox}$ mice were 82% heavier than controls injected with EGFP only (*PVH*$^{Cre/+}$;*Scn9a*$^{flox/flox}$: 198.5% ± 15.8% versus *PVH*$^{+/+}$;*Scn9a*$^{flox/flox}$: 116.5% ± 2.7% of pre-injection body weight; t test, p = 0.007). Therefore, PVH neurons in vivo require Na$_V$1.7 for efficient integration of sparse synaptic input to reliably reach action potential threshold and to regulate body weight.

DISCUSSION

Collectively, these experiments show a synaptic integration mechanism dependent on Na$_V$1.7 that selectively extends excitatory synaptic potentials in several hypothalamic neuron populations and is critical for maintaining energy homeostasis. Each cell type that we examined with high expression of *Scn9a* (AGRP, POMC, and PVH neurons) exhibited near-perfect synaptic input integration. These hypothalamic neurons show step-like changes in membrane potential in response to synaptic input,

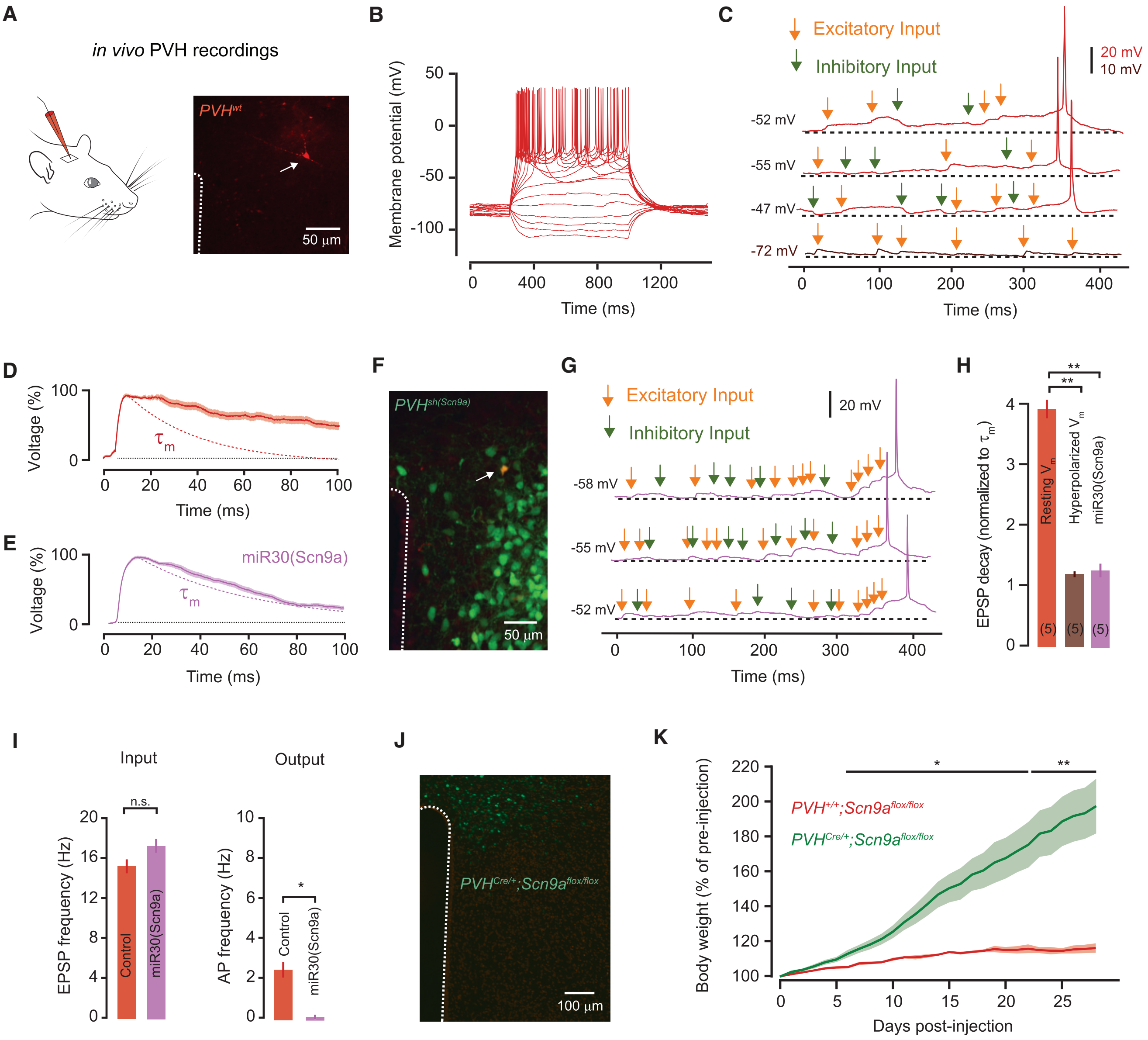

Figure 7. Near-Perfect Synaptic Integration in PVH Neurons In Vivo

(A) Schematic of in vivo recordings in the PVH in anesthetized mice (left), and image (right) of biocytin filled PVH cell recovered after whole-cell recording (arrow).

(B) Example voltage response to current step injections.

(C) Voltage recording of spontaneous activity showing that the interaction between excitatory and inhibitory inputs in vivo generates prolonged EPSPs and step-wise integration preceding action potential firing. Somatic hyperpolarization removes the EPSP prolongation (bottom trace).

(D) Example of peak-scaled average spontaneous EPSP for one cell showing marked prolongation beyond the membrane time constant (τ_m, dashed line).

(E) Knockdown of *Scn9a* in *Sim1*^{Cre} mice with *hrGFP-miR30(Scn9a)* abolishes EPSP prolongation in vivo (τ_m, dashed line).

(F) Image of a PVH cell recovered after in vivo whole-cell recording in a *Sim1*^{Cre} mouse expressing *hrGFP-miR30(Scn9a)* in the PVH (green cells). The recorded cell (arrow) is stained for biocytin in red as in (A) and is EGFP-positive, and thus appears yellow.

(G) *Scn9a* knockdown abolishes near-perfect integration in vivo

(H) Summary data for the effect of hyperpolarization and *Scn9a* knockdown on the EPSP decay time.

(I) *Scn9a* knockdown disrupt the in vivo input-output conversion.

(J) Image of Cre-EGFP expressing cells in the PVH of a *Scn9a*$^{flox/flox}$ mouse.

(K) Daily body weight after Cre-EGFP (n = 8) or EGFP (n = 4) targeting to the PVH of *Scn9a*$^{flox/flox}$ mice showing rapid development of obesity in *PVH*$^{Cre/+}$*; Scn9a*$^{flox/flox}$ mice. Bar graphs or lines with shaded areas show mean ± SEM.

See also Figure S5.

which allows extraordinarily efficient summation of excitatory synaptic potentials. In contrast, VMH neurons had low *Scn9a* expression and showed conventional leaky input integration. Importantly, deep-brain in vivo whole-cell recordings demonstrated that PVH neurons receive sparse excitatory synaptic inputs with step-like voltage changes preceding neuron firing, confirming that this mode of synaptic integration is used in the mouse brain. Thus, these experiments reveal a specialization of hypothalamic neurons that set behavioral state over extended timescales to prolong synaptic inputs for near-perfect integration.

Loss of $Na_v1.7$ in AGRP, POMC, and PVH neurons resulted in significant body weight changes that were consistent in magnitude with previous studies that have interfered with signaling pathways in these neuron populations (Balthasar et al., 2004; Tolson et al., 2010; van de Wall et al., 2008). In these hypothalamic populations, the most prominent neuronal effect of reduced $Na_v1.7$ expression was diminished synaptic integration due to loss of persistent sodium current. Notably, action potential threshold and amplitude as well as synaptic release were unchanged with reduction of $Na_v1.7$, which may be due to compensation from multiple other voltage gated sodium channels expressed in these neurons. Thus, the loss of near-perfect synaptic input integration reduces the output of these cell types and leads to altered body weight set-point. Although the effect on body weight may also be related to additional aspects of $Na_v1.7$ function in these neurons, the prominent effect on synaptic integration indicates the importance of this efficient integration property in vivo for the role of these hypothalamic cell types in energy homeostasis.

Why do some hypothalamic neuron populations show near-perfect synaptic integration, while other populations do not? Efficient synaptic integration has several important consequences (Knight, 1972; Pressley and Troyer, 2009). First, it can generate firing with temporally sparse inputs in a linear manner. This may be an important property for neural circuits that regulate or encode motivational drives, as it generates a constant rate of output as a function of a particular level of synaptic input. Second, it produces poorly timed spikes, suggesting that the information is transmitted to downstream circuits as a rate code. This view is supported by the fact that AGRP neurons elicit feeding behavior with a variety of firing patterns, and the output of AGRP neurons is a strikingly prolonged asynchronous release of GABA (Aponte et al., 2011; Atasoy et al., 2012; Krashes et al., 2011). Third, prolonged EPSPs increase sensitivity to low frequency inputs, which may be required in AGRP, POMC, and PVH neurons to provide continuous influence on appetite and body weight. These properties of AGRP, POMC, and PVH neurons contrast with cortical or possibly even VMH circuits (Lin et al., 2011), which influence behavior on very rapid timescales and would be adversely affected by lack of temporal precision and coincidence detection.

Although it has been noted that all voltage-gated sodium channels can elicit persistent currents (Taddese and Bean, 2002), our experiments indicate that, in these hypothalamic neurons, $Na_v1.7$ plays a more specialized role for prolonging synaptic input. This function is not solely a property of $Na_v1.7$ expression. A computational model based on experimentally determined AGRP neuron conductances can produce prolonged EPSPs, but this requires a narrow range of parameters. To permit stable, stepwise increases in membrane potential, I_{NaP} and voltage-sensitive potassium channel conductances must be appropriately tuned along with high input resistance and a depolarized resting membrane potential. One implication of the interplay between these conductances is that efficient synaptic integration in hypothalamic neurons might be a sensitive control point for regulation of neuron activity by sodium and potassium channel gene expression as well as neuromodulation. In addition, not all synaptic inputs were prolonged in neurons showing efficient integration. The decay time for $\sim$20% of the EPSPs in AGRP neurons was not greater than the membrane time constant (Figure 1C). This might indicate input-specific processing of voltage changes from excitatory synapses. One possible explanation is that the distribution of $Na_v1.7$ channels or, alternatively key potassium conductances necessary for prolonged input integration, might be differentially localized at specific neuronal compartments such as to subsets of dendritic branches or spines. In addition, our observation that transient sodium current amplitude and action potential threshold were not significantly altered by ablation or knockdown of *Scn9a* could reflect dendritic localization of $Na_v1.7$ in these cells or upregulation of other sodium channels in response to loss of *Scn9a*. Additional studies will be required to establish the role of subcellular sodium and potassium channel distributions for efficient excitatory synaptic integration in these hypothalamic populations.

Notably, $Na_v1.7$ has been primarily studied for its role in pain, and antagonism of $Na_v1.7$ is being investigated for anti-nociception therapies. However, our demonstration of widespread $Na_v1.7$ distribution in the hypothalamus and its critical involvement in energy homeostasis functions show a new role for this ion channel and also indicate that pharmacological therapeutic strategies targeting $Na_v1.7$ in the peripheral nervous system might preferrably avoid action in the central nervous system.

Efficient synaptic integration in AGRP, POMC, and PVH neurons reveals the important role of synaptic control over the electrical activity of these cell types, which has previously been primarily associated with direct hormonal regulation. Recent studies have shown multiple sources of excitatory synaptic control over AGRP, POMC, and other hypothalamic neurons, as well as prominent synaptic plasticity (Atasoy et al., 2012; Krashes et al., 2014; Liu et al., 2012; Pinto et al., 2004; Sternson et al., 2005; Yang et al., 2011). We show here that these neurons have specialized neuronal computation characteristics due to $Na_v1.7$ expression. In light of the striking differences in synaptic input integration that we observed in distinct hypothalamic neuron populations, synaptic integration properties are an essential consideration for understanding hypothalamic neural circuit function.

EXPERIMENTAL PROCEDURES

Fluorescent In Situ Hybridization

Two-color fluorescence in situ hybridization (FISH) was performed on hypothalamus-containing fixed frozen sections from male *Agrp*^{Cre} mice (8–9 weeks

old), using the proprietary probes and methods of Advanced Cell Diagnostics (see the Supplemental Experimental Procedures).

Constructs for *Scn9a* Knockdown

miR30-based shRNA constructs for *Scn9a* were developed using miR_Scan software (http://www.ncbi.nlm.nih.gov/staff/ogurtsov/projects/mi30/) (Matveeva et al., 2012) with the *Scn9a* coding sequence (NM_01290674.1, position: 3728-3748). To produce a negative control for this *miR30*-based *Scn9a* shRNA construct, we used a website to produce a scrambled sequence (http://www.sirnawizard.com/scrambled.php) and then chose a sequence with <76% homology to RefSeq transcripts in the mouse genome and that also obeyed guidelines for *miR30*-based shRNA (Dow et al., 2012; Matveeva et al., 2012) (see the Supplemental Experimental Procedures).

Electrophysiology in Brain Slices

Acute coronal slices (200 μm) were prepared at the level of the ARC from male mice (5–10 weeks) expressing a viral vector or fluorescent proteins in AGRP or POMC neurons (see the Supplemental Experimental Procedures).

Pharmacology

Recordings of EPSCs and intracellular voltage were done in the presence of picrotoxin. Measurements of firing rate using cell-attached recordings were done without blockers (see the Supplemental Experimental Procedures).

Modeling

All simulations were performed with the NEURON simulation environment (Hines and Carnevale, 1997). Passive parameters were C_m = 1 μF/cm^2, R_i = 100 Ω.cm, R_m = 7,000 Ω.cm^2 for single compartment and R_m = 35,000 Ω.cm^2 for the multi-compartmental model, adjusted to match experimental values of input resistance and membrane time constant. Active conductances were (in millisiemens (mS)/cm^2): voltage-activated fast sodium channels (axon 100, soma 100; single compartment 0), voltage-activated potassium channels (axon 10, soma 5; single compartment 3.5; V_{half} adjusted, to match the experimentally recorded potassium current-voltage relationship). I_{NaP} was modeled as an activating and non-inactivating Na^+ current: $I_{NaP} = g_{NaP} * m * (V - V_{Na})$, as previously described (Uebachs et al., 2010) with V_{half} adjusted to −35 mV and placed in the soma at a density of 0.065 mS/cm^2, matching the current density recorded in AGRP neurons (0.0478 mS/cm^2 for single compartment). AMPA receptor-mediated synaptic conductances were modeled as a double exponential function (τ_{rise} = 0.5 ms, τ_{decay} = 1.0 ms, g_{max} = 0.5 nanosiemens [nS]). Voltage-clamp was simulated as a SEClamp process with R_s = 10 MΩ. Simulation files will be made available at the ModelDB at http://senselab.med.yale.edu/modeldb/.

Statistics

Values are represented as mean ± SEM p values for pairwise comparisons were calculated using SciPy or SigmaPlot. We used paired and unpaired two-tailed Student's t tests for normally distributed data (tested with the Shapiro-Wilk test) with equal variance (tested with the Levene test) and Mann-Whitney U tests when these conditions were violated. All comparisons for *Scn9a* shRNA are done against scrambled shRNA unless otherwise noted. For all measurements, values with scrambled shRNA in AGRP or POMC neurons were not significantly different from control Npy^{hrGFP} or $Pomc^{topazFP}$, respectively (n.s. $p > 0.05$, *$p < 0.05$, **$p < 0.01$, ***$p < 0.001$).

SUPPLEMENTAL INFORMATION

Supplemental Information includes Supplemental Experimental Procedures and five figures and can be found with this article online at http://dx.doi.org/10.1016/j.cell.2016.05.019.

AUTHOR CONTRIBUTIONS

T.B. and S.M.S. conceived the project and prepared the manuscript with comments from all authors. T.B., A.T., and C.J.M. performed the ex vivo electrophysiology. C.J.M. and A.T. performed in vivo recordings with technical assistance from S.T. and A.K.L. T.B. analyzed the electrophysiology data and made the computational model. S.M.S. designed shRNA constructs. K.S. acquired RNA-seq data for AGRP neurons. J.N.W. provided key reagents.

ACKNOWLEDGMENTS

This research was funded by the Howard Hughes Medical Institute (to S.M.S.), the HHMI Janelia Research Campus Visitor program (to T.B.), the NeuroSeq Project Team (to K.S.), a Wellcome Trust/Royal Society Henry Dale Fellowship (098400/Z/12/Z) and Medical Research Council (MRC) grant MC-UP-1201/1 (to T.B.), and MRC grant G091905 and Wellcome Trust Grant 10154 (to J.N.W.). We thank H. Su for molecular biology; M. Copeland for RNA-FISH; K. Ritola for virus production; A. Zeladonis and S. Lindo for mouse breeding, genotyping, and histology; L. Jin for mouse weight measurements; and S. Eddy, B. Clark, M. Hausser, A. Roth, and C. Schmidt-Hieber for comments on the manuscript. This paper is dedicated to Professor Stuart L. Schreiber on the occasion of his 60th birthday.

Received: May 11, 2015
Revised: March 27, 2016
Accepted: April 26, 2016
Published: June 16, 2016

REFERENCES

Aponte, Y., Atasoy, D., and Sternson, S.M. (2011). AGRP neurons are sufficient to orchestrate feeding behavior rapidly and without training. Nat. Neurosci. *14*, 351–355.

Atasoy, D., Betley, J.N., Su, H.H., and Sternson, S.M. (2012). Deconstruction of a neural circuit for hunger. Nature *488*, 172–177.

Balthasar, N., Coppari, R., McMinn, J., Liu, S.M., Lee, C.E., Tang, V., Kenny, C.D., McGovern, R.A., Chua, S.C., Jr., Elmquist, J.K., and Lowell, B.B. (2004). Leptin receptor signaling in POMC neurons is required for normal body weight homeostasis. Neuron *42*, 983–991.

Branco, T., and Häusser, M. (2011). Synaptic integration gradients in single cortical pyramidal cell dendrites. Neuron *69*, 885–892.

Carter, B.C., Giessel, A.J., Sabatini, B.L., and Bean, B.P. (2012). Transient sodium current at subthreshold voltages: activation by EPSP waveforms. Neuron *75*, 1081–1093.

Catterall, W.A. (2000). From ionic currents to molecular mechanisms: the structure and function of voltage-gated sodium channels. Neuron *26*, 13–25.

Cox, J.J., Reimann, F., Nicholas, A.K., Thornton, G., Roberts, E., Springell, K., Karbani, G., Jafri, H., Mannan, J., Raashid, Y., et al. (2006). An SCN9A channelopathy causes congenital inability to experience pain. Nature *444*, 894–898.

Cummins, T.R., Howe, J.R., and Waxman, S.G. (1998). Slow closed-state inactivation: a novel mechanism underlying ramp currents in cells expressing the hNE/PN1 sodium channel. J. Neurosci. *18*, 9607–9619.

Dib-Hajj, S.D., Yang, Y., Black, J.A., and Waxman, S.G. (2013). The Na(V)1.7 sodium channel: from molecule to man. Nat. Rev. Neurosci. *14*, 49–62.

Dow, L.E., Premsrirut, P.K., Zuber, J., Fellmann, C., McJunkin, K., Miething, C., Park, Y., Dickins, R.A., Hannon, G.J., and Lowe, S.W. (2012). A pipeline for the generation of shRNA transgenic mice. Nat. Protoc. *7*, 374–393.

Farries, M.A., Kita, H., and Wilson, C.J. (2010). Dynamic spike threshold and zero membrane slope conductance shape the response of subthalamic neurons to cortical input. J. Neurosci. *30*, 13180–13191.

French, C.R., Sah, P., Buckett, K.J., and Gage, P.W. (1990). A voltage-dependent persistent sodium current in mammalian hippocampal neurons. J. Gen. Physiol. *95*, 1139–1157.

Fricker, D., and Miles, R. (2000). EPSP amplification and the precision of spike timing in hippocampal neurons. Neuron *28*, 559–569.

Gao, Q., and Horvath, T.L. (2007). Neurobiology of feeding and energy expenditure. Annu. Rev. Neurosci. *30*, 367–398.

Gulledge, A.T., Kampa, B.M., and Stuart, G.J. (2005). Synaptic integration in dendritic trees. J. Neurobiol. *64*, 75–90.

Henry, F.E., Sugino, K., Tozer, A., Branco, T., and Sternson, S.M. (2015). Cell type-specific transcriptomics of hypothalamic energy-sensing neuron responses to weight-loss. eLife *4*, e09800.

Hines, M.L., and Carnevale, N.T. (1997). The NEURON simulation environment. Neural Comput. *9*, 1179–1209.

Iansek, R., and Redman, S.J. (1973). The amplitude, time course and charge of unitary excitatory post-synaptic potentials evoked in spinal motoneurone dendrites. J. Physiol. *234*, 665–688.

Jack, J.J.B., Noble, D., and Tsien, R.W. (1975). Electric Current Flow in Excitable Cells (Oxford University Press).

Knight, B.W. (1972). Dynamics of encoding in a population of neurons. J. Gen. Physiol. *59*, 734–766.

König, P., Engel, A.K., and Singer, W. (1996). Integrator or coincidence detector? The role of the cortical neuron revisited. Trends Neurosci. *19*, 130–137.

Krashes, M.J., Koda, S., Ye, C., Rogan, S.C., Adams, A.C., Cusher, D.S., Maratos-Flier, E., Roth, B.L., and Lowell, B.B. (2011). Rapid, reversible activation of AgRP neurons drives feeding behavior in mice. J. Clin. Invest. *121*, 1424–1428.

Krashes, M.J., Shah, B.P., Madara, J.C., Olson, D.P., Strochlic, D.E., Garfield, A.S., Vong, L., Pei, H., Watabe-Uchida, M., Uchida, N., et al. (2014). An excitatory paraventricular nucleus to AgRP neuron circuit that drives hunger. Nature *507*, 238–242.

Lin, D., Boyle, M.P., Dollar, P., Lee, H., Lein, E.S., Perona, P., and Anderson, D.J. (2011). Functional identification of an aggression locus in the mouse hypothalamus. Nature *470*, 221–226.

Liu, T., Kong, D., Shah, B.P., Ye, C., Koda, S., Saunders, A., Ding, J.B., Yang, Z., Sabatini, B.L., and Lowell, B.B. (2012). Fasting activation of AgRP neurons requires NMDA receptors and involves spinogenesis and increased excitatory tone. Neuron *73*, 511–522.

Magee, J.C. (2000). Dendritic integration of excitatory synaptic input. Nat. Rev. Neurosci. *1*, 181–190.

Matveeva, O.V., Nazipova, N.N., Ogurtsov, A.Y., and Shabalina, S.A. (2012). Optimized models for design of efficient miR30-based shRNAs. Front. Genet. *3*, 163.

Pinto, S., Roseberry, A.G., Liu, H., Diano, S., Shanabrough, M., Cai, X., Friedman, J.M., and Horvath, T.L. (2004). Rapid rewiring of arcuate nucleus feeding circuits by leptin. Science *304*, 110–115.

Prescott, S.A., and De Koninck, Y. (2005). Integration time in a subset of spinal lamina I neurons is lengthened by sodium and calcium currents acting synergistically to prolong subthreshold depolarization. J. Neurosci. *25*, 4743–4754.

Pressley, J., and Troyer, T.W. (2009). Complementary responses to mean and variance modulations in the perfect integrate-and-fire model. Biol. Cybern. *101*, 63–70.

Schmalhofer, W.A., Calhoun, J., Burrows, R., Bailey, T., Kohler, M.G., Weinglass, A.B., Kaczorowski, G.J., Garcia, M.L., Koltzenburg, M., and Priest, B.T. (2008). ProTx-II, a selective inhibitor of NaV1.7 sodium channels, blocks action potential propagation in nociceptors. Mol. Pharmacol. *74*, 1476–1484.

Shadlen, M.N., and Newsome, W.T. (1998). The variable discharge of cortical neurons: implications for connectivity, computation, and information coding. J. Neurosci. *18*, 3870–3896.

Spruston, N. (2008). Pyramidal neurons: dendritic structure and synaptic integration. Nat. Rev. Neurosci. *9*, 206–221.

Stegmeier, F., Hu, G., Rickles, R.J., Hannon, G.J., and Elledge, S.J. (2005). A lentiviral microRNA-based system for single-copy polymerase II-regulated RNA interference in mammalian cells. Proc. Natl. Acad. Sci. USA *102*, 13212–13217.

Stern, P., Astrof, S., Erkeland, S.J., Schustak, J., Sharp, P.A., and Hynes, R.O. (2008). A system for Cre-regulated RNA interference in vivo. Proc. Natl. Acad. Sci. USA *105*, 13895–13900.

Sternson, S.M., Shepherd, G.M., and Friedman, J.M. (2005). Topographic mapping of VMH–> arcuate nucleus microcircuits and their reorganization by fasting. Nat. Neurosci. *8*, 1356–1363.

Stuart, G., and Sakmann, B. (1995). Amplification of EPSPs by axosomatic sodium channels in neocortical pyramidal neurons. Neuron *15*, 1065–1076.

Taddese, A., and Bean, B.P. (2002). Subthreshold sodium current from rapidly inactivating sodium channels drives spontaneous firing of tuberomammillary neurons. Neuron *33*, 587–600.

Tolson, K.P., Gemelli, T., Gautron, L., Elmquist, J.K., Zinn, A.R., and Kublaoui, B.M. (2010). Postnatal Sim1 deficiency causes hyperphagic obesity and reduced Mc4r and oxytocin expression. J. Neurosci. *30*, 3803–3812.

Uebachs, M., Opitz, T., Royeck, M., Dickhof, G., Horstmann, M.T., Isom, L.L., and Beck, H. (2010). Efficacy loss of the anticonvulsant carbamazepine in mice lacking sodium channel beta subunits via paradoxical effects on persistent sodium currents. J. Neurosci. *30*, 8489–8501.

van de Wall, E., Leshan, R., Xu, A.W., Balthasar, N., Coppari, R., Liu, S.M., Jo, Y.H., MacKenzie, R.G., Allison, D.B., Dun, N.J., et al. (2008). Collective and individual functions of leptin receptor modulated neurons controlling metabolism and ingestion. Endocrinology *149*, 1773–1785.

Van Wart, A., and Matthews, G. (2006). Impaired firing and cell-specific compensation in neurons lacking nav1.6 sodium channels. J. Neurosci. *26*, 7172–7180.

Vega, A.V., Henry, D.L., and Matthews, G. (2008). Reduced expression of Na(v) 1.6 sodium channels and compensation by Na(v)1.2 channels in mice heterozygous for a null mutation in Scn8a. Neurosci. Lett. *442*, 69–73.

Wang, D., He, X., Zhao, Z., Feng, Q., Lin, R., Sun, Y., Ding, T., Xu, F., Luo, M., and Zhan, C. (2015). Whole-brain mapping of the direct inputs and axonal projections of POMC and AgRP neurons. Front. Neuroanat. *9*, 40.

Weiss, J., Pyrski, M., Jacobi, E., Bufe, B., Willnecker, V., Schick, B., Zizzari, P., Gossage, S.J., Greer, C.A., Leinders-Zufall, T., et al. (2011). Loss-of-function mutations in sodium channel Nav1.7 cause anosmia. Nature *472*, 186–190.

Williams, S.R., and Stuart, G.J. (2000). Site independence of EPSP time course is mediated by dendritic I(h) in neocortical pyramidal neurons. J. Neurophysiol. *83*, 3177–3182.

Yang, Y., Atasoy, D., Su, H.H., and Sternson, S.M. (2011). Hunger states switch a flip-flop memory circuit via a synaptic AMPK-dependent positive feedback loop. Cell *146*, 992–1003.

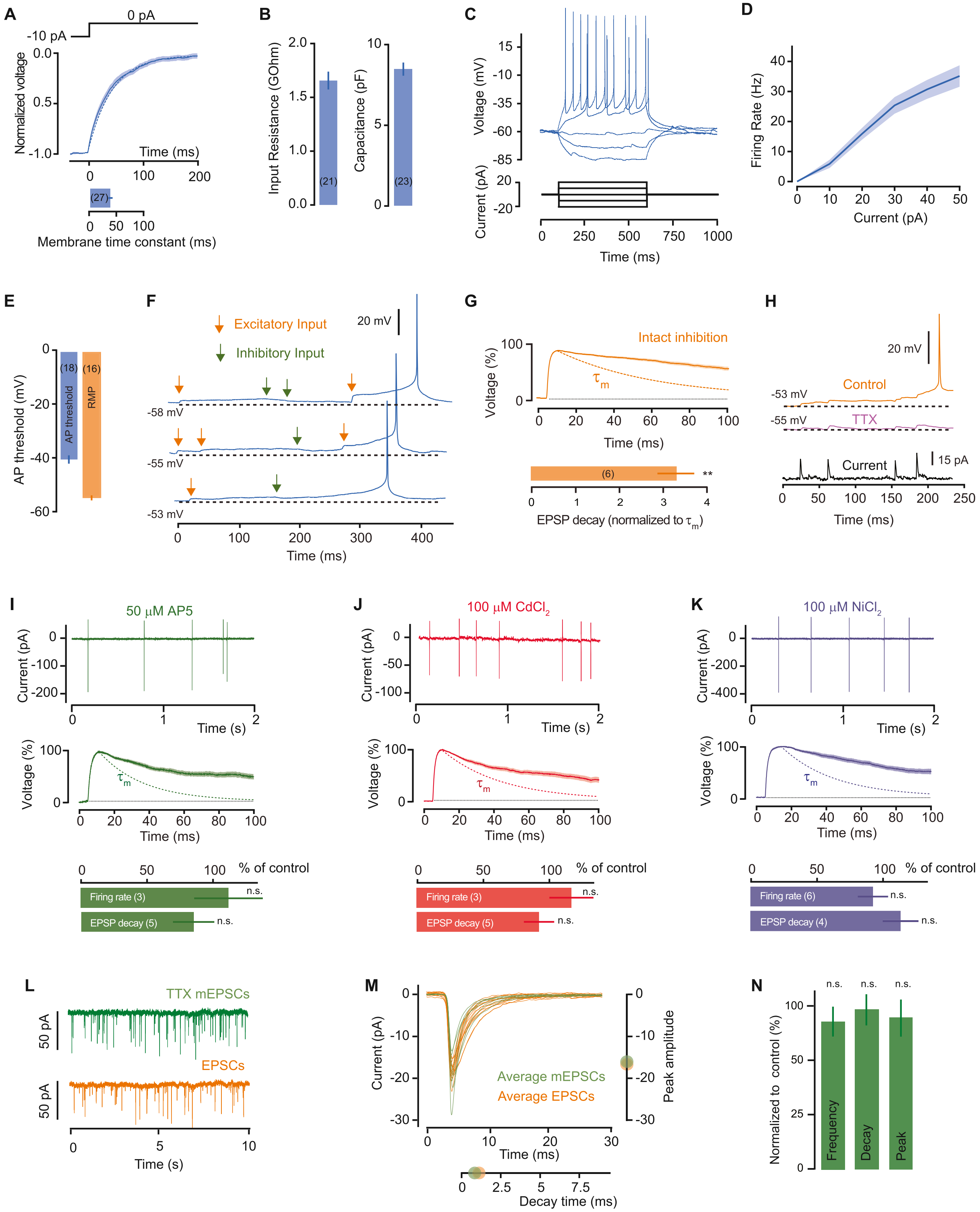

(legend on next page)

Figure S1. Core Biophysical Properties of AGRP Neurons and Pharmacology of Synaptic Integration, Related to Figure 1

(A) Estimation of the membrane time constant of AGRP neurons, by fitting a single exponential (dashed line) to the membrane potential decay after a hyperpolarizing current step (trace shows average across cells).

(B) Mean input resistance (1.5 ± 0.1 GΩ) and capacitance (8.5 ± 0.4 pF) of AGRP neurons.

(C) Example voltage response to current step injections.

(D) Mean current-firing rate relationship for all cells.

(E) AP threshold (−41.2 ± 1.2 mV) and resting membrane potential (RMP, −55.6 ± 1.3 mV). Mean ± SEM.

(F) Voltage recording of spontaneous activity in AGRP neurons showing that step-wise integration of excitatory input can overcome synaptic inhibition to reach AP threshold.

(G) Top, average peak scaled EPSP for one cell with synaptic inhibition intact (τ_m: dashed orange line) and summary data (bottom) showing that EPSP decays remain significantly slower than the membrane time constant, despite the presence of synaptic inhibition (3.3 ± 0.4 of τ_m; paired t test, $p = 0.004$).

(H) Injection of recorded mESPC waveforms reproduces step-wise integration, which is abolished by TTX (227 ± 22% of τ_m, n = 4 for control versus 99.0 ± 6%, n = 7 for TTX; unpaired t test, $p = 0.0001$).

(I–K) Blocking NMDA receptors (I), L-type (J) and T-type (K) voltage-gated calcium channels has no significant effect on the firing rate (unpaired t tests versus *Npy*^{hrGFP} cells, AP5: $p = 0.71$, $CdCl_2$: $p = 0.48$, $NiCl_2$: $p = 0.46$) or EPSP kinetics of AGRP neurons (unpaired t tests versus *Npy*^{hrGFP} cells, AP5: $p = 0.37$, $CdCl_2$: $p = 0.80$, $NiCl_2$: $p = 0.20$). τ_m: colored dashed lines. Traces in the top and middle rows are examples for individual cells, and bottom row shows summary data for all cells. Samples sizes in parentheses.

(L) Example traces for spontaneous EPSCs and mEPSCs recorded at −70 mV.

(M) Average EPSC and mEPSC waveforms for individual cells with the population mean peak amplitude (right) and mean decay time (below).

(N) Summary data for mEPSC comparisons against EPSCs (frequency: U-test, $p = 0.47$ n = 7; decay time: unpaired t test, $p = 0.73$, n = 7; peak amplitude: unpaired t test, $p = 0.48$, n = 8 TTX). Bar graphs or lines with shaded areas show mean ± SEM n.s. $p > 0.05$, **$p < 0.01$.

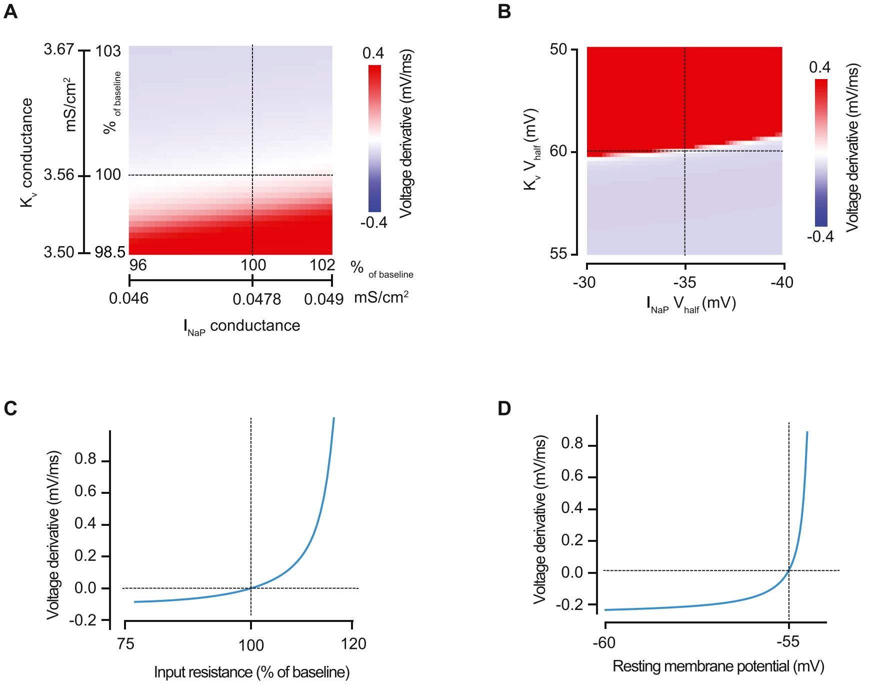

Figure S2. EPSP Prolongation in a Single Compartment Model Depends on the Properties of I_{NaP} and K_v, Input Resistance, and Membrane Potential, Related to Figure 2

(A) Plot of the mean EPSP derivative (first 50 ms) as a function of I_{NaP} and K_v conductance densities (similar to Figure 2C), shows that stable voltage levels are only achieved for a narrow ratio of I_{NaP} and K_v conductance values (white band, where the mean derivative is close to 0 mV/ms).

(B) Stable EPSP prolongation shows a sharp dependency on the I_{NaP} and K_v half-activation voltages (V_{half}). Hyperpolarized I_{NaP} and depolarized K_v activation voltages produce EPSPs that quickly lead to action potentials (red area), whereas the inverse lead to fast decaying EPSPs (blue area).

(C) Changing the model input resistance by ± 20% severely disrupts EPSP prolongation.

(D) In the model, optimal resting membrane potential for EPSP prolongation is −55 mV. Hyperpolarized potentials fail to activate enough I_{NaP} and more depolarized values engage a strong positive feedback loop that leads to action potentials. Dashed lines indicate optimal values.

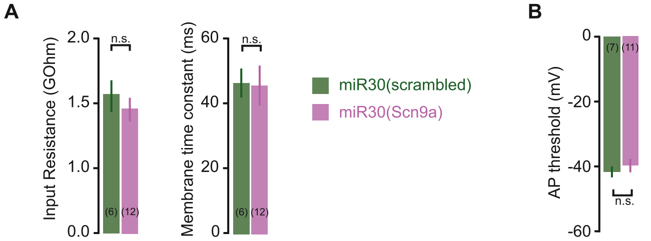

Figure S3. *miR30-Scn9a* Does Not Change Basic AGRP Neurons Biophysical Properties, Related to Figure 5

(A) Input resistance and membrane time constant are not significantly different between *miR30(Scn9a)* and *miR30(scrambled-Scn9a)*. Input resistance, $AGRP^{sh(Scn9a\text{-}scram)}$:1.4 ± 0.2 GΩ; $AGRP^{sh(Scn9a)}$: 1.3 ± 0.1 GΩ; Membrane time constant, $AGRP^{sh(Scn9a\text{-}scram)}$: 46.5 ± 4.5 ms; $AGRP^{sh(Scn9a)}$: 45.5 ± 6.1 ms.

(B) The voltage threshold for action potential initiation is also not significantly affected by *miR30(Scn9a)*. $AGRP^{sh(Scn9a\text{-}scram)}$: −43.5 ± 0.9 mV; $AGRP^{sh(Scn9a)}$: −40.0 ± 1.2 mV; Mean ± SEM. Samples sizes in parentheses. Data are represented as mean ± SEM n.s. $p > 0.05$.

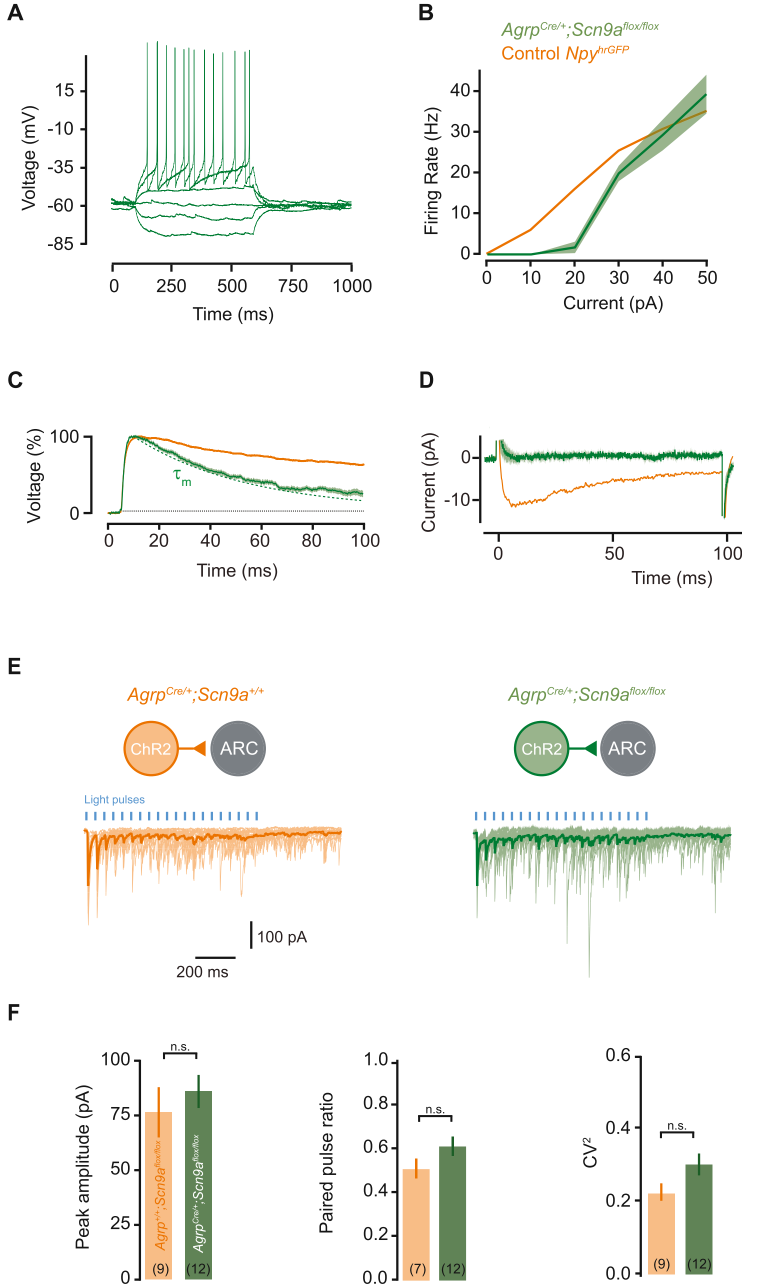

Figure S4. Electrophysiological Properties of AGRP Neurons with *Scn9a* Knockout, Related to Figure 6

(A) Example voltage response to current step injections in *Agrp*$^{Cre/+}$;*Scn9a*$^{flox/flox}$ cells, identified for recording by expression of *rAAV2/9-CAG-FLEX-EGFP*.

(B) Mean current-firing relationship showing an increase in the rheobase in comparison to control *Npy*^{hrGFP} cells (89.2 ± 9% lower firing rate for 20 pA steps compared to *Npy*^{hrGFP}; n = 6, U-test, $p < 0.01$), but the peak firing rate is similar.

(C) Example average EPSP for one cell (green), showing no prolongation beyond the membrane time constant (decay = 104.2 ± 10% of τ_m, n = 4, paired t test, p = 0.76). Dashed line: τ_m. Orange, data from control *Npy*^{hrGFP} cells.

(D) *Scn9a* knockout abolishes the persistent current in response to a +5 mV depolarizing voltage step (current at 50 ms = 5.7 ± 5% of control *Npy*^{hrGFP} cells, n = 5, U-test, $p < 0.001$).

(E) *Scn9a* knockout does not affect postsynaptic inhibitory currents generated in arcuate neurons by Channelrhodopsin stimulation of AGRP neurons (left, control; right, *Scn9a* knockout). Traces are from examples cells, light colors are individual trials and dark trace is the average response.

(F) Summary data showing that peak IPSC amplitude and two measures of presynaptic function, paired-pulse ratio and the squared coefficient of variation are not affected by *Scn9a* knockout in AGRP neurons. Bar graphs or lines with shaded areas show mean ± SEM n.s. $p > 0.05$.

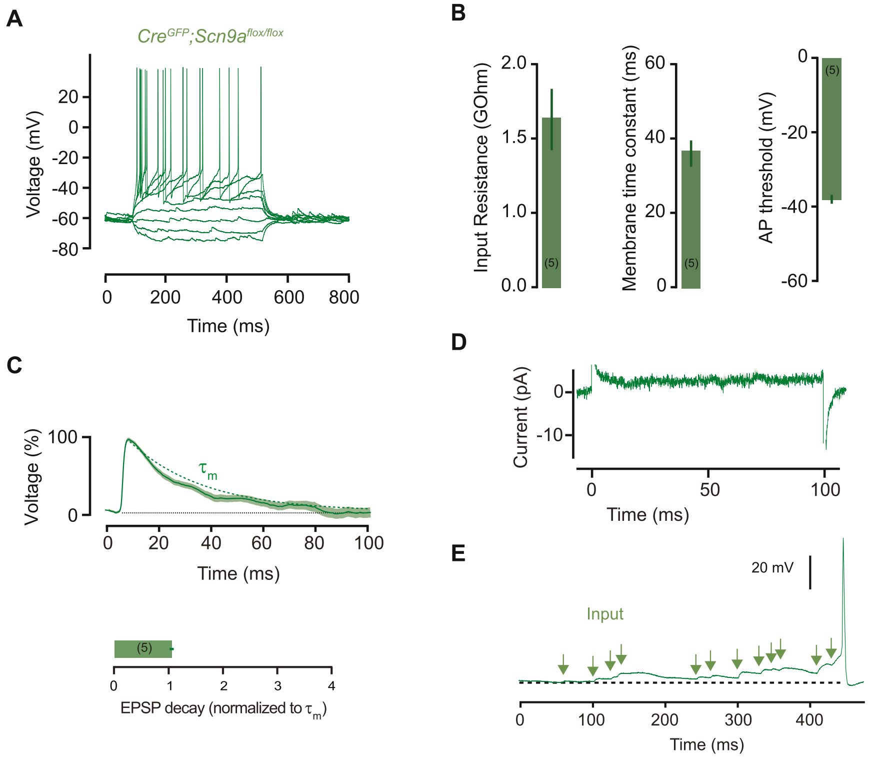

Figure S5. Electrophysiological Properties of PVH Neurons with *Scn9a* Deletion, Related to Figure 7

(A) Example voltage response to current step injections in *Scn9a*$^{flox/flox}$ cells expressing *rAAV2/9-CAG-Cre-EGFP*.

(B) Mean input resistance (1.4 ± 0.3 GΩ), membrane time constant (37.5 ± 3.7 ms) and AP threshold (−37.6 ± 1.1 mV) of PVH neurons with *Scn9a* knockout.

(C) Example average EPSP for one *Scn9a* knockout cell decaying as predicted by membrane time constant (decay = 104 ± 4% of τ_m, n = 5, paired t test, p = 0.44). Dashed line: τ_m.

(D) Average response of all cells to a +5 mV depolarizing voltage step, showing no persistent inward current (current at 50 ms = +3.0 ± 0.6 pA).

(E) Synaptic integration is disrupted by *Scn9a* knockout (c.f. Figure 3J). Bar graphs or lines with shaded areas show mean ± SEM.

Cell, Volume *165*

Supplemental Information

Near-Perfect Synaptic Integration by Na_v1.7 in Hypothalamic Neurons Regulates Body Weight

Tiago Branco, Adam Tozer, Christopher J. Magnus, Ken Sugino, Shinsuke Tanaka, Albert K. Lee, John N. Wood, and Scott M. Sternson

Supplemental Experimental Procedures

All experimental protocols were conducted according to U.S. National Institutes of Health guidelines for animal research and approved by the Institutional Animal Care and Use Committee at Janelia Research Campus. Experiments conducted in the UK were licensed under the UK Animals (Scientific Procedures) Act of 1986 following local ethical approval.

Mice. Mice were housed on a 06:00-18:00h light cycle with water and mouse chow *ad libitum* (PicoLab Rodent Diet 20, 5053 tablet, TestDiet) unless otherwise noted. Adult male mice (>6 weeks old) were used for experiments. Cre recombinase-expressing lines were used: Npy^{hrGFP} (Jackson Labs Stock 006417), $Pomc^{topazFP}$ (Jackson Labs Stock 008322), $Agrp^{Cre}$ (Jackson Labs Stock 012899, $Agrp^{tm1(cre)Lowl}$/J), $Pomc^{Cre}$ (BAC transgenic provided by Dr. Joel Elmquist, University of Texas Southwestern Medical Center), $Scn9a^{tm1.1Jnw}$ ($Scn9a^{flox/flox}$, J.N.W., University College London) (Weiss et al., 2011). To generate $Agrp^{Cre/+;}Scn9a^{flox/flox}$ or $Pomc^{Cre/+};Scn9a^{flox/flox}$, homozygous $Scn9a^{flox/flox}$ mice were crossed to either $Agrp^{Cre}$ or $Pomc^{Cre}$ and the double heterozygous progeny were then crossed with homozygous $Scn9a^{flox/flox}$. $Agrp^{Cre/+;}Scn9^{flox/flox-}$ or $Pomc^{Cre/+;}Scn9a^{flox/flox}$ mice were identified by genotyping tail DNA; in addition the possibility for global *Scn9a* deletion was monitored by including a primer for the presence of the knock-out allele in the genotyping PCR reaction, which was negative for all mice included in the study. For tail DNA from a subset of animals we also sequenced the genotyping band and confirmed that the loxP flanked exons 14 and 15 of *Scn9a* were intact.

Recombinant adeno-associated viral (rAAV) vectors. The following viral vectors (Atasoy et al., 2008) were used in this study: rAAV2/9-*CAG::FLEX-rev-hrGFP:miR30(Scn9a)* and rAAV2/9-*CAG::FLEX-rev-hrGFP:miR30(scrambled-Scn9a)* (1.5 e13 - 1.7e13 Genomic Copies (GC)/ml, Janelia, http://www.addgene.org/Scott_Sternson/), rAAV2/9-*CAG*-FLEX-EGFP (7e12 GC/ml, Penn), *rAAV9.hSyn.HI.eGFP-Cre* (7e12GC/ml,Penn), *rAAV9.eF1a.dflox.ChR2(H134R).mCherry*

(7e12 GC/ml, Penn) *CAG*: promoter containing a cytomegalovirus enhancer; chicken beta actin promoter, first exon, and first intron; and the splice acceptor of rabbit beta-globin gene. FLEX: Cre-dependent flip-excision switch (Atasoy et al., 2008).

Viral injections were performed as described previously (Betley et al., 2013). Bilateral ARC viral injections in *Agrp*^{Cre} *or Pomc*^{Cre} mice were made at two depths using the following coordinates: bregma: -1.7 mm; midline: ±0.24 mm; dorsal surface: -5.95 mm (100 nl/site). Coordinates for bilateral PVH injection in *Scn9a*$^{flox/flox}$ mice were: bregma -0.85mm; midline -0.22mm; dorsal surface -4.77mm (40 nl/site). Mice were used for electrophysiology 8-12 days post infection.

RNA sequencing. AGRP neurons were manually sorted from the ARC of *Npy*^{hrGFP} mice using methods described previously (Hempel et al., 2007). The full RNA-Seq data set has been recently reported (Henry et al., 2015).

Fluorescent in situ hybridization. Two-color FISH was performed on hypothalamus-containing fixed frozen sections from male *Agrp*^{Cre} mice (8-9 weeks old), using the proprietary probes and methods of Advanced Cell Diagnostics (ACD Technical notes #320535 for tissue prep, and #320293 for Multiplex labeling, http://www.acdbio.com/technical-support/downloads). Briefly animals were anesthetized and sequentially perfused with RNase free solutions of PBS and 4% paraformaldehyde (PFA) in phosphate buffered saline (PBS). The brains were removed and post-fixed (24 h, 4 ºC) in 4% PFA in PBS, incubated in 30% sucrose (12 h), and the blocked brain was mounted in cryo-embedding media (OCT) on a cryostat for sectioning. Frozen sections (15 µm) were mounted on slides, which were air dried (20 min at -20 ºC) or stored -80 ºC for later use. The OCT was washed off with PBS before pretreatment with ACD proprietary reagents PT2 & PT4. After boiling for 5 min in PT2, sections were rinsed in distilled water then ethanol, air dried, and then incubated with ACD proprietary reagent PT4 (30 min, 40 ºC) in a

HybEZ sealed humidified incubator (Advanced Cell Diagnostics). We performed dual probe labeling, using probes for *Scn9a* in Channel 1 (Mm-Scn9-C1, #313341-C1), and either *Agrp* (Mm-Agrp-C2, #400711-C2) or *Pomc* (Mm-Pomc-C2, #314081-C2) in Channel 2. Probes were mixed at a 1:50 ratio of Channel 2 and Channel 1 probes. Wax-outlined tissue sections were immersed in Probe mix and incubated (2 h, 40 ºC) in the HybEZ humidified incubator, rinsed in ACD Wash Buffer (2 x 2') then sequentially incubated in ACD proprietary reagents alternating AMP1-FL and AMP3-FL (30 min) with AMP2-FL and AMP4-FL (15 min) with 2 washes (2 min) between each step. Brain sections were then labeled with DAPI and coverslips were applied. Slides were stored at 4 ºC before image acquisition at 10×, 20×, and 63× using a Zeiss 710 confocal on an Axio Examiner Z1 upright microscope. Quantification of *Scn9a* particles (Fig. 2F) was performed on single confocal optical sections acquired with a 63× objective, using StarSeach (http://rajlab.seas.upenn.edu/StarSearch/launch.html) with a threshold setting of 50. For colocalization of *Scn9a* with *Agrp* or *Pomc*, we used single confocal optical sections (acquired with a 63× objective); for the small number of cells that did not show colocalization in a single optical section AGRP: (3/328 neurons, POMC: 2/169 neurons) we examined other optical sections for the neuron and classified as co-expressing if we identified *Scn9a* elsewhere in the neuron (colocalization detected in 3/5 of these neurons).

Constructs for *Scn9a* knockdown. *miR30*-based shRNA constructs for *Scn9a* were developed using miR_Scan software (http://www.ncbi.nlm.nih.gov/staff/ogurtsov/projects/mi30/) (Matveeva et al., 2012) with the *Scn9a* coding sequence (NM_01290674.1, position: 3728-3748). Using miR_Scan and associated rules for *miR30*-based shRNA (Dow et al., 2012; Matveeva et al., 2012), we selected the sense strand sequence CGGGUGGCAUACGGGUAUAAAA (based on design rules (Dow et al., 2012), the 5' base of shRNA sense strand is not homologous to the coding sequence) and the guide strand sequence UUUUAUACCCGUAUGCCACCCA (miR_Scan score: 84.6, sequence position: 3727-3748). As

an additional criterion, we looked for homology to mouse RefSeq RNA <80%, and for established RefSeq mRNA in the mouse, the closest homology sequences, other than *Scn9a*, were Olfr372 (61% identity) and Traf3 (57% identity). A *miR30* construct based on previously described sequence (Stern et al., 2008) was synthesized with sense and guide strand sequences from *Scn9a* and was placed in the 3'UTR of a hrGFP expression vector in pcDNA3.1. To produce a negative control for this *miR30*-based *Scn9a* shRNA construct, we used an online tool to produce a scrambled sequences (http://www.sirnawizard.com/scrambled.php) and then chose a sequence with <76% homology to RefSeq transcripts in the mouse genome and that also obeyed guidelines for *miR30*-based shRNA (Dow et al., 2012; Matveeva et al., 2012) (sense strand: ACGAAGTCAGGGAGGAATTTAT; guide strand: AUAAAUUCCUCCCUGACUUCGC). The sequences were subcloned into pcDNA3.1. After establishing effective knockdown of *Scn9a* in HEK cells (see below) with *hrGFP-miR30(Scn9a)* but not *hrGFP-miR30(scambled-Scn9a)*, we placed these sequences into an rAAV vector between a FLEX switch sequence (Atasoy et al., 2008) downstream of a *CAG* promoter in an rAAV2 vector.

HEK cell electrophysiology. Constructs for shRNA were characterized in whole cell voltage clamp experiments performed on HEK293 cells stably expressing murine *Scn9a*. Cells were plated at low density ($4\times10^3/cm^2$) on poly-D-lysine coated glass coverslips in 10 cm^2 wells, then transfected using the Fugene 6 transfection reagent (Roche) with a DNA plasmid (pcDNA3.1) with *hrGFP-miR30(Scn9a)* or *hrGFP-miR30(scrambled-Scn9a)* (2 μg per well), followed by replacement of media after 3 h. Recordings were made 24-72 h post-transfection. Patch clamp recordings were made 24-72 h post-transfection in whole cell voltage clamp on an inverted amplifier (Molecular Devices CA). Electrophysiological data was filtered at 5 kHz, digitized at 20 kHz using a Digidata 1400 interface (Molecular Devices CA), recorded on a PC (Superlogics), and analysed using custom Matlab software (MathWorks). Electrode resistance was 2-3 MOhm and 85-95% series resistance compensation was routinely applied. Cells were continuously

perfused from a gravity fed perfusion system. External solution contained (mM): NaCl 140, HEPES 10, Glucose 5, KCl 4, $CaCl_2$ 2, $MgCl_2$ 1. Whole cell pipette internal solution contained (mM); CsCl 31, CsGluconate 87, NaCl 5, HEPES 10, EGTA 10 MgATP 3. Osmolarity was within 280-300 mOsm/L, and pH was adjusted using CsOH to within 7.3 - 7.4.

Electrophysiology in brain slices. Acute coronal slices (200 μm) were prepared at the level of the ARC from male mice (5-10 weeks) expressing a viral vector or fluorescent proteins in AGRP or POMC neurons. Animals were killed by decapitation following isoflurane anaesthesia. Coronal slices were cut at 4°C using a 7000smz-2 vibrating microtome (Campden, UK). Brain slices were incubated at 37°C for one hour before being kept at room temperature prior to experiments, in artificial cerebrospinal fluid (ACSF) containing (in mM): $NaCl_2$ 125, KCl 2.5, $NaHCO_3$ 26, NaH_2PO_4 1.25, glucose 25, $CaCl_2$ 2, $MgCl_2$ 1 (pH 7.3 when bubbled with 95% O_2 and 5% CO_2). Boroscillicate glass micropipettes with a 3-6 MΩ resistance (Harvard Apparatus, UK) were filled with (in mM): K-Gluconate 130, KCl 10, HEPES 10, EGTA 1, Na_2ATP 2, Mg_2ATP 2, Na_2GTP 0.3, filtered (2 μm) prior to patching. For cell-attached recordings pipettes were filled with ACSF and currents recorded in voltage-clamp with a holding current of 0 pA. EPSCs were recorded at a holding potential of -70 mV. Transient sodium currents were elicited with a 50 ms voltage step to 0 mV from -80 mV. Fluorescent cells were visualized on an upright Slicescope (Scientifica, UK) using a 60× objective. Somatic whole-cell patch clamp recordings (R_{series} <30 MΩ) were achieved using a HEKA 800 Amplifier (HEKA, Germany). Data was acquired at 25-50 kHz using custom software and filtered at 8 kHz. All voltage recordings were obtained with a seal resistance > 5 GΩ and within 10 minutes of gaining whole-cell access, and cells were discarded if there was more than a 10% change in input resistance. All recordings were performed at 35-37°C.

***In vivo* electrophysiology.** *In vivo* whole cell patch clamp recordings, were made from control and virally transduced *miR30(Scn9a)* knockdown in *Sim1*^{Cre} mice in the PVH. Mice were

sequentially anesthetized with 2% isoflurane, which was replaced by a longer-term intraperitoneal anesthetic mix, comprised of Fentanyl (0.0075 mpk), Dexdomitor (0.112 mpk), and Midazolam (6 mpk), which was supplemented with doses of 1/3 the initial volume every 45 minutes (Lee et al., 2012). Mice were secured in a stereotaxic frame, local anesthetic was applied (marcaine, 2 mg/kg), a small (diameter <1.0 mm) craniotomy was drilled in the skull over the PVH (~-0.8 mm A/P and +0.2 mm M/L) and the dura was removed.

Due to the physical resistances involved in traversing almost 5 mm of brain with a patch pipette in this region, we found that best results were obtained by perforating a pathway to 4.6 mm below the surface of the cortex, nearly to the level of the PVH, which was followed by introducing the recording pipet via this lower resistance pathway. The perforation was performed with a patch electrode similar to those used for recordings, but broken back to 50 µm diameter at the tip and sharpened by beveling at ~35°. This pipette was filled with the same HEPES buffered ACSF used in our HEK cell recordings. No positive air pressure was applied to the pipette during perforation.

In vivo whole-cell recordings were made after using positive air pressure to maintain a clear pipette tip during descent (surface→-4.5 mm: 900 mB, -4.5 mm→-4.6 mm: 200 mB, -4.6 mm→-4.7 mm: 50 mB, then 15-20 mB while stepping at 1 µm steps through the PVH (PVH was at -4.7 mm to -4.9 mm from cortical surface, calibrated with fluorescent bead injections in pilot experiments) until contact with a cell was detected by a change in the pipette tip resistance, and a gigaseal formed. Patch electrodes (4-10 MOhm) contained 125 mM Potassium Gluconate, 11 mM Potassium Chloride, 10 mM HEPES, 10 mM Phosphocreatine, 4 mm ATP, 0.3 mM GTP, and 2.5 mM Biocytin, pH 7.3 and 285 mOsm. Data was recorded on a HEKA Patchmaster system, sampled at 50 kHz after filtering at 10 kHz.

After whole-cell recordings *in vivo* we obtained outside-out patches to ensure membrane re-sealing and processed the tissue for biocytin labeling. Mice were returned to deep isoflurane anesthesia, and perfused with PBS then 4% PFA in phosphate buffered saline (PBS).

Vibratome sections (60 µm) were cut coronally from whole brains embedded in agarose. Biocytin fills were labeled in free floating slices by overnight incubation at 4°C in PBS containing 0.01% Triton X-100 and Alexa594 conjugated streptavidin (1 µg/ml, Life Technologies), then washed thoroughly (12-24 h in PBS with 3 changes of solution) in the same wells, before being mounted on slides using Vectashield and imaged using a 10× objective on a Zeiss LSM510 confocal inverted microscope. All recordings included in the dataset were associated with labeled cells in the PVH, though for some recordings it was not possible to unambiguously match biocytin-labeled PVH neurons to the recorded cell due to extensive uptake of biocytin by multiple PVH neurons. For all recordings after shRNA *Scn9a* knock-down, biocytin labeling was observed in PVH neurons expressing the shRNA construct. However because of extensive biocytin uptake we could not unambiguously assign every recording to these neurons and thus analyzed all PVH neurons recorded from shRNA transduced mice as the *Scn9a* knock-down group.

Channelrhodopsin-assisted circuit mapping. Acute coronal slices were prepared from $Agrp^{Cre/+};Scn9a^{flox/flox}$ mice or $Agrp^{Cre/+};Scn9a^{wt/wt}$ mice (>7 weeks old) injected with *rAAV9.eF1a.dflox.ChR2(H134R)*. After a minimum of 2 weeks post-injection, whole-cell recordings were made from non-fluorescent cells in the arcuate nucleus, and IPSCs were recorded upon wide-field stimulation with 1 ms 488 nm light pulses delivered at 20 Hz.

Pharmacology. Recordings of EPSCs and intracellular voltage were done in the presence of Picrotoxin, except for the data shown in Figure S1F-G. Measurements of firing rate using cell-attached recordings were done without blockers. Drugs were bath applied with a Miniplus 3 (Gilson, FR) peristaltic pump perfusion system. Blockers and final concentrations were: Glutamate receptors (AP-5 50 µM, Tocris; Kynurenic Acid 2 mM, Sigma; NBQX 1 µM Tocris), $GABA_A$ receptors (Picrotoxin 50 µM, Sigma); Na_V channels (TTX 1µM, Tocris), K_V channels (TEA 10 mM, Sigma); Ca_V channels ($CdCl_2$ 100µM and $NiCl_2$ 50 µM; Sigma); $Na_v1.7$ (Protoxin-II

2 nM, Alomone Labs). Unless otherwise noted, slices were perfused in blockers at least 10 min prior to obtaining recordings, and all comparisons made are against the population of cells recorded in the absence of blockers.

Data analysis. Electrophysiology data was analyzed in Python 2.7 using custom written routines. The values for I_{NaP} reported in the text and in Figures 1 and 5 are for peak currents at -30 mV obtained with ramp protocols recorded in the presence of blockers of voltage-gated potassium and calcium channels (TEA, $CdCl_2$ and $NiCl_2$), and with the leak subtracted by fitting a linear function to the initial, linear part of the current response. In cells that passed the stability criteria, TTX was applied and the peak TTX-sensitive current directly measured in the same cell (e.g., Figure 1G), which was not significantly different from the above procedure. Membrane time constants were estimated for each cell by fitting a single exponential to the decay of a hyperpolarizing current step (-10 pA). EPSPs and IPSPs were detected automatically by threshold algorithm applied to the first derivative of the voltage traces. Values of EPSP decay times normalized to the membrane time constant were obtained by measuring the fractional decay from the EPSP at 100 ms after the EPSP onset, and dividing by the decay predicted by membrane time constant on a cell-by-cell basis. All membrane potential values reported have been corrected for junction potentials.

Supplemental References

Atasoy, D., Aponte, Y., Su, H.H., and Sternson, S.M. (2008). A FLEX switch targets Channelrhodopsin-2 to multiple cell types for imaging and long-range circuit mapping. J Neurosci *28*, 7025-7030.

Betley, J.N., Cao, Zhen Fang H., Ritola, Kimberly D., and Sternson, Scott M. (2013). Parallel, Redundant Circuit Organization for Homeostatic Control of Feeding Behavior. Cell *155*, 1337-1350.

Dow, L.E., Premsrirut, P.K., Zuber, J., Fellmann, C., McJunkin, K., Miething, C., Park, Y., Dickins, R.A., Hannon, G.J., and Lowe, S.W. (2012). A pipeline for the generation of shRNA transgenic mice. Nat Protoc *7*, 374-393.

Hempel, C.M., Sugino, K., and Nelson, S.B. (2007). A manual method for the purification of fluorescently labeled neurons from the mammalian brain. Nat Protoc *2*, 2924-2929.

Henry, F.E., Sugino, K., Tozer, A., Branco, T., and Sternson, S.M. (2015). Cell type-specific transcriptomics of hypothalamic energy-sensing neuron responses to weight-loss. Elife *4:e09800*.
Lee, D., Lin, B.J., and Lee, A.K. (2012). Hippocampal place fields emerge upon single-cell manipulation of excitability during behavior. Science *337*, 849-853.
Matveeva, O.V., Nazipova, N.N., Ogurtsov, A.Y., and Shabalina, S.A. (2012). Optimized models for design of efficient miR30-based shRNAs. Frontiers in genetics *3*, 163.
Stern, P., Astrof, S., Erkeland, S.J., Schustak, J., Sharp, P.A., and Hynes, R.O. (2008). A system for Cre-regulated RNA interference in vivo. Proc Natl Acad Sci U S A *105*, 13895-13900.
Weiss, J., Pyrski, M., Jacobi, E., Bufe, B., Willnecker, V., Schick, B., Zizzari, P., Gossage, S.J., Greer, C.A., Leinders-Zufall, T.*, et al.* (2011). Loss-of-function mutations in sodium channel Nav1.7 cause anosmia. Nature *472*, 186-190.
